# The Stiffness‐Sensitive Transcriptome of Human Tendon Stromal Cells

**DOI:** 10.1002/adhm.202101216

**Published:** 2023-01-20

**Authors:** Amro A. Hussien, Barbara Niederoest, Maja Bollhalder, Nils Goedecke, Jess G. Snedeker

**Affiliations:** ^1^ Institute for Biomechanics ETH Zurich Zurich 8092 Switzerland; ^2^ Balgrist University Hospital University of Zurich Zurich 8008 Switzerland

**Keywords:** connective tissues, fibroblasts, matrix stiffness, mechanobiology, soft matter, tendons

## Abstract

Extracellular matrix stiffness is a major regulator of cellular states. Stiffness‐sensing investigations are typically performed using cells that have acquired “mechanical memory” through prolonged conditioning in rigid environments, e.g., tissue culture plastic (TCP). This potentially masks the full extent of the matrix stiffness‐driven mechanosensing programs. Here, a biomaterial composed of 2D mechanovariant silicone substrates with simplified and scalable surface biofunctionalization chemistry is developed to facilitate large‐scale cell culture expansion processes. Using RNA sequencing, stiffness‐mediated mechano‐responses of human tendon‐derived stromal cells are broadly mapped. Matrix elasticity (*E*.) approximating tendon microscale stiffness range (*E*. ≈ 35 kPa) distinctly favors transcriptional programs related to chromatin remodeling and Hippo signaling; whereas compliant stiffnesses (*E*. ≈ 2 kPa) are enriched in processes related to cell stemness, synapse assembly, and angiogenesis. While tendon stromal cells undergo dramatic phenotypic drift on conventional TCP, mechanovariant substrates abrogate this activation with tenogenic stiffnesses inducing a transcriptional program that strongly correlates with established tendon tissue‐specific expression signature. Computational inference predicts that AKT1 and ERK1/2 are major stiffness‐sensing signaling hubs. Together, these findings highlight how matrix biophysical cues may dictate the transcriptional identity of tendon cells, and how matrix mechano‐reciprocity regulates diverse sets of previously underappreciated mechanosensitive processes in tendon fibroblasts.

## Graphical abstract

Extracellular matrix stiffness‐sensing studies are typically carried out using cells that have acquired “mechanical memory” through progressive passaging in rigid mechanical environments, e.g., tissue culture plastic (TCP). This confounds the mechanistic experiments that investigate matrix stiffness‐driven mechanosensing programs. This work reports the formulation of a mechanovariant silicone‐based platform with tunable elasticity and tendon‐like elastic (*E*.) moduli, and with optimized chemistry to make it amenable to large‐scale cell expansion practices.

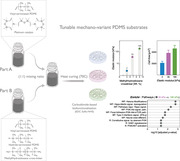

## 1 Introduction

Aberrant extracellular matrix (ECM) stiffening is a feature of progressive aging, and is associated with several chronic inflammatory and metabolic disorders.^[^ ^1^ ^]^ Stiffened microenvironments in which tendon stromal cells reside significantly differ from that of healthy tissues, with a growing body of evidence suggesting that matrix stiffness cues govern key cellular processes in tendon.^[^ ^2^ ^]^ Extracellular matrix stiffening is a driver of persistent activation of connective tissue stromal fibroblasts.^[^ ^3^ ^]^ Resident stromal cells secret matrix constituents during tissue development and sustain it during homeostasis. In turn, extracellular matrix niches relay active and passive biophysical cues that are transduced intracellularly into biochemical signals via mechanotransduction machineries.^[^ ^4^ ^]^ Elevate matrix stiffness acts as a feed‐forward activation loop which enhances fibro‐inflammatory activation of stromal cells leading to excessive tissue remodeling and further stiffening of the extracellular matrix.^[^ ^3^, ^5^ ^]^ How resident fibroblasts sense and integrate matrix stiffness cues is a fertile area for investigation.

Standard tissue culture plastic (TCP) vessels have been routinely used for adherent cell culture for over five decades.^[^ ^6^ ^]^ Although TCP surfaces are optically clear and facilitate cell adhesion in vitro, they possess supra‐physiological levels of stiffness with elastic (*E*.) modulus in the range of Gigapascals (GPa). In contrast, most connective tissues have cell‐scale matrix stiffness with *E*. modulus values in the kilopascal (kPa) range, which may increase by up to two orders of magnitude in case of fibrosis.^[^ ^2^, ^7^ ^]^ It is now widely appreciated that progressive passaging on TCP mechanically activates stromal fibroblasts in vitro and instills an epigenetically‐imprinted mechanical memory that is retained over the long term.^[^ ^8^ ^]^

Numerous biomaterial systems have been developed to closely mimic optimal tissue stiffness.^[^ ^9^ ^]^ Previous mechanobiology studies have largely harnessed polyacrylamide hydrogels or Sylgard‐based silicone substrates in small to medium scales that were limited to experimental work.

Although polyacrylamide hydrogels have been the most widely employed substrates in rigidity‐sensing investigations, they require complex, multistep protocols to facilitate hydrogels bonding to glass slides or to conjugate ECM proteins to its surfaces. Furthermore, increasing the polyacrylamide stiffnesses by varying the crosslinking density simultaneously alters hydrogel pore size and potentially the stochastic spacing of ECM ligands.^[^ ^10^ ^]^ Alternatively, silicone‐based substrates are traditionally fabricated using conventional Polydimethylsiloxane (PDMS) Sylgard kits. Sylgard 184 formulation was originally designed as a filler to insulate sensitive electrical/electronic circuits, and it contains high amounts of silica nanoparticle impurities.^[^ ^11^ ^]^ This significantly contributes to its batch‐to‐batch variability and reproducibility issues, especially when Sylgard 184 parts are mixed in nonstoichiometric ratios (e.g., 80:1 or 100:1) to produce cultures substrates at the softer end of the spectrum (≈1–2 kPa).^[^ ^12^ ^]^ There is clearly a need for an in vitro system that enables rigidity‐sensing mechanotransduction studies, while seamlessly integrating within standard cell culture practices to facilitate large‐scale cell expansion in tissue‐like physiological stiffnesses.

Here, we introduce a mechanovariant silicone‐based platform with tunable stiffness spanning a wide range of connective tissue‐like *E*. moduli (2–180 kPa). We used this system to map the stiffness‐induced transcriptional mechano‐responses and unveiled previously undescribed processes that are involved in stiffness‐sensing in tendon‐derived stromal cells.

## 2 Results

### 2.1 Tunable Mechanovariant PDMS Substrates for Mechanobiology

**Figure 1:**
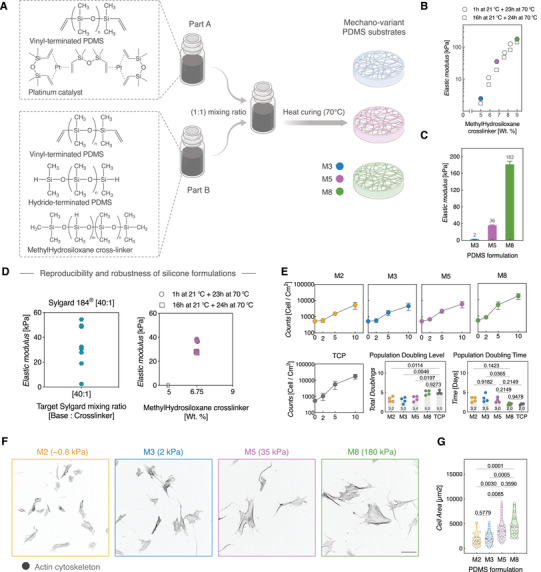
Tunable mechano‐variant PDMS substrates for mechanobiology. The silicone formulations cover a wide spectrum of elasticities from very soft to stiff. A) Schematic illustration of the constituent components of the PDMS culture substrates. Recipes were formulated so that the optimal polymerization conditions are initiated by mixing Part A and Part B at 1:1 mixing ratio, while tuning the target *E*. modulus only by varying the amounts of methylhydrosiloxane copolymer cross‐linker in Part B. B) Material characterization of substrates mechanical properties (*E*. modulus) for the indicated PDMS formulations, as measured by micro‐indentation using a calibrated piezoresistive probe. The stiffness is dependent on the methylhydrosiloxane cross‐linker concentration and curing conditions. C) PDMS stiffness ranges used in this study. Bars represent the mean ± SD of the closed symbols in figure (B). D) Reproducibility of *E*. modulus of [40:1] Sylgard 184 and M5 PDMS formulation. *N* = (8 samples, Sylgard 184) and (6 samples, M5 formulation). E) Cell proliferation, population doubling level (PDL), and population doubling time (PDT) of mechano‐variant PDMS substrates compared to tissue culture plastic (TCP). (*N* = 4 independent biological replicates, i.e., different donors). F,G) Substrate stiffness impacts tendon stromal cells morphology on mechano‐variant substrates. F) Representative images of human tendon stromal cells on mechano‐variant PDMS substrates. Actin cytoskeleton (Gray). Scale bar = 100 µm. G) Quantification of single cell spreading area in different mechano‐variant substrates. *N* = 16–21 cells from two independent biological donors with two independent biological donors.

To overcome the limitations associated with using conventional mechano‐culture substrates, we developed a tunable Polydimethylsiloxane (PDMS)‐based mechanovariant culture system using pure, room temperature vulcanizing (RTV) silicone. We started from vinyl‐terminated PDMS, hydride‐terminated PDMS, and methylhydrosiloxane copolymer cross‐linker which reacts in the presence of a platinum catalyst (**Figure** **1**A). We stoichiometrically balanced the silicone precursors to yield two separate components (Part A and Part B) that start to polymerize when mixed at a (1:1) mixing ratio. This design criterion is especially critical for the consistent reproducibility of soft substrates (<5 kPa), which is a bottleneck when off‐the‐shelf Sylgard 184 kits are used. Since the silicone mixture precursors start reacting immediately when Part A and Part B get in contact, we further optimized the time between mixing the components and heat‐curing the mixture in order to tune the target stiffness of the bulk material (Figure 1B). Additionally, we copolymerized a fixed amount of undecenoic acid to graft carboxyl groups in the PDMS surface, thus providing tethering points for covalent conjugation of the primary amines of ECM proteins. This step facilitates the use of EDC‐NHS amine‐reactive crosslinker to couple collagen type I (or any other ECM molecule) to the PDMS substrates. Ultimately, we successfully produced mechanovariant silicone substrates with elastic moduli (*E*.) ranging from 2 to 180 kPa, spanning the previously reported tenogenic stiffness range (≈20–40 kPa), the tissue‐level stiffness of acutely torn tendons (≈1–3 kPa) and the stiffness of granulation tissue of torn tendons during healing (≈160–200 kPa) (Figure 1C).^[^ ^13^ ^]^

To demonstrate the robustness and reproducibility of our formulation, we compared the *E*. modulus of the M5 formulation with [40:1] Sylgard 184 mix, targeting an *E*. value of ≈35 kPa. While Sylgard mixes exhibited considerable variability in the measured *E*. moduli, our M5 formulation showed robust reproducibility in the two hot curing protocols that have been optimized in this work, indicating improved mechanical accuracy and precision of our formulation compared to off‐the‐shelf Sylgard 184 silicone (Figure 1D and Figure S1, Supporting Information).

Next, we quantitatively characterized the proliferation of tendon stromal cells on the mechanovariant substrates. We monitored the temporal changes in cell numbers over 10 d. Cell proliferation was measured by counting the number of cells at days 2, 5, and 10. All the mechanovariant substrates supported the initiation and exponential growth of tendon cells (Figure 1E). Cell population doubling level (PDL) and population doubling time (PDT) for the soft to intermediated stiffnesses (M2, M3, and M5) ranged between 3–3.4 doublings and 3–3.5 d, respectively, with no statistically significant difference between the conditions. In contrast, cells cultured on stiff (M8) substrates showed significantly higher PDL and shorter PDT that were comparable to conventional TCP cultures (Figure 1E). Finally, we cultured tendon stromal cells for 24 h at low seeding density (i.e., ≈250 cells cm^‐2^) to quantify cell spreading areas. Cells adopted spread morphologies across all stiffnesses, with significantly larger cell areas in the intermediate (M5, ≈35 kPa) and rigid (M8, 180 kPa) stiffnesses relative to the softer conditions (Figure 1F,G).

### 2.2 Transcriptomic Comparison of Stiffness‐Sensing in Human Tendon Stromal Cells

**Figure 2:**
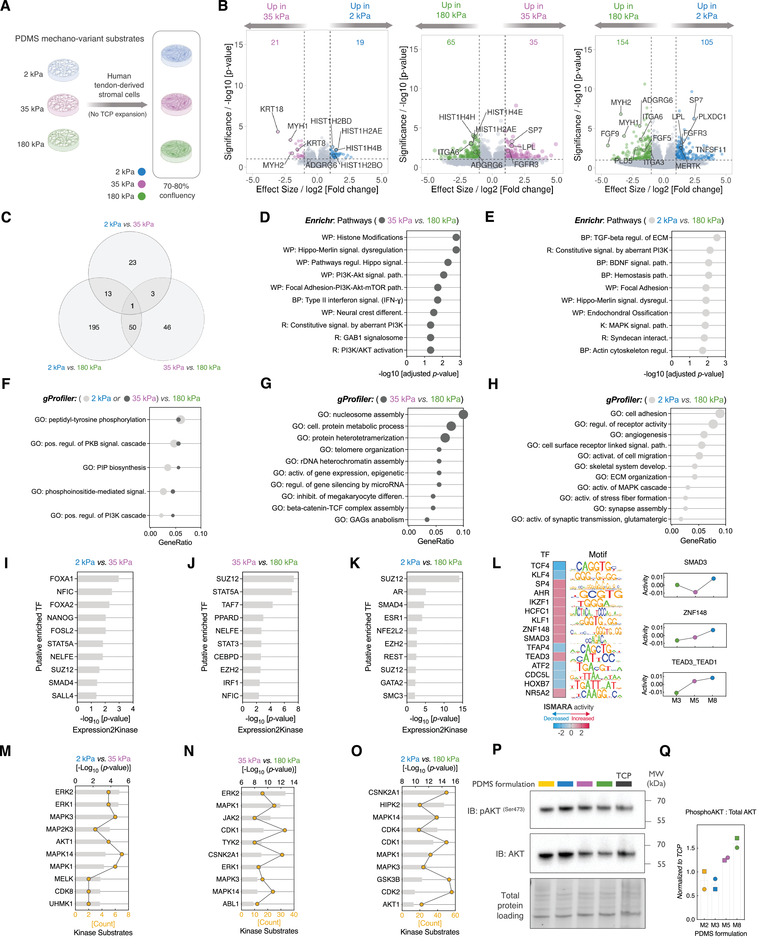
Transcriptomic comparison of stiffness‐sensing in human tendon stromal cells. A) Schematic overview of the range of substrate stiffnesses that were used to interrogate stiffness‐sensing in this study. Healthy human tendon‐derived cells were isolated from patients who underwent autograft tendon transfer for the repair of knee anterior cruciate ligament (ACL). Freshly dissociated cells were directly seeded on PDMS substrates without the standard expansion on tissue culture plastics. B) RNA‐seq volcano plots of DEGs across different stiffness comparisons. Differential expression was determined by *DESeq2* method. Colored values represent significant DEGs, which are defined with cut‐off values of |fold change| ± 1 and *p*‐value < 0.01. C) Venn diagram depicting the overlapped significantly expressed genes (DEGs) between the different stiffness comparisons. D,E) Top 10 enriched pathways for each stiffness comparison; D) 35 kPa versus 180 kPa, E) 2 kPa versus 180 kPa. Enrichment analysis was performed in *Enrichr* using both up‐regulated and down‐regulated genes. DEGs were interrogated against BioPlanet 2019 (BP), Reactome (R), WikiPathways 2019 (WP), KEGG 2019 (K) databases. The list of significantly enriched pathways was manually curated, and it is listed according to the smallest *p*‐values obtained across all the databases. F) Shared enriched biological processes (BP) GO terms between the indicated stiffness comparisons using *gProfiler* over‐representation analysis of both upregulated and downregulated DEGs. G,H) Top enriched biological processes (BP) GO terms in each stiffness comparison; G) 35 kPa versus 180 kPa, H) 2 kPa versus 180 kPa. Overrepresentation analysis was performed using hypergeometric overrepresentation test against the GO database with both upregulated and downregulated DEGs. Significance achieved at *q‐*value < 0.05. I–K) Bar plots show the predicted top 10 most significantly enriched transcription factors upstream of the DEGs. Predicted TFs are sorted by significance level (adjusted *p*‐value < 0.05), and were inferred using the TF Enrichment Analysis modules of the *Expression2Kinase* tool. L) ISMARA inferred regulatory motif analysis of predicted TFs activity at proximal promoter regions, as a function of the substrate stiffness. Dot plots depict ISMARA‐predicted activity of SMAD3, ZNF148, AND TEAD3‐TEAD1 motifs. M–O) Bar plots indicate the predicted top 10 most significantly enriched kinases upstream of the DEGs. Predicted kinases are ranked by significance level (adjusted *p*‐value < 0.05). Kinases were predicted using the Kinase Enrichment Analysis modules of the *Expression2Kinase* tool. P) Representative immunoblot and quantification of Phospho‐Akt^(Ser473)^ kinase. Data are from two independent biological donors. All data is shown as fold change to TCP.

To address the role of matrix stiffness in shaping early transcriptional responses of “naïve” tendon‐derived stromal cells, we seeded acutely isolated cells directly on collagen type I‐coated PDMS substrates at three different stiffnesses: soft (2 kPa), intermediate (35 kPa) and rigid (180 kPa) (**Figure** **2**A). We have previously shown that intermediate stiffnesses (20–40 kPa) favored tenogenic differentiation of bone marrow mesenchymal stromal cells in an ECM ligand‐dependent manner.^[^ ^13^, ^14^ ^]^ Here, tendon‐derived stromal cells were not expanded on TCP and were maintained in culture until reaching 70–80% confluency. Details on clinical information of all donors are included in Table S1 (Supporting Information).

We first conducted pairwise differential expression analysis which revealed progressive increase in the number of differentially expressed genes (DEGs) at the extreme of stiffness comparisons (2 vs 180 kPa: 259 DEGs) relative to the comparisons within narrower ranges (2 kPa vs 35 kPa: 40 DEGs or 35 kPa vs 180kPa: 100 DEGs) (Figure 2B,C). DEGs showed minimal overlapping across all the stiffness comparisons suggesting that stiffness‐mediated transcriptional responses are unique within each stiffness range (Figure 2C). Using *Enrichr*, we next searched for the stiffness‐responsive pathways that are functionally enriched in the upregulated and down‐regulated DEGs.^[^ ^15^ ^]^ We found that the transcriptome of stromal cells on the (35 vs 180 kPa) range was enriched in pathways known to be mechanosensitive or mechanically regulated, such as chromatin and histone modifications, Hippo‐Merlin pathway, and the PI3K‐Akt pathway (Figure 2D). In contrast, cells on the (2 vs 180 kPa) comparison were transcriptionally enriched in a wider range of pathways related to extracellular matrix (TGF‐beta regulation of ECM, syndecan interactions), endochondral ossification, cell adhesion and cytoskeleton (Figure 2E).

Of note is that the PI3K‐Akt pathway and its related processes were consistently enriched across the two stiffness comparisons, suggesting it might be a key signaling hub regulating the stiffness‐sensitive responses in tendon stromal fibroblasts (Figure 2F). Examining the enriched pathways in the stiffness‐specific genes, i.e., DEGs exclusively expressed in one stiffness range but not the others, revealed that histone modifications and TGF‐beta regulation of ECM were the most significantly enriched pathways in the (35 vs 180 kPa) and (2 vs 180 kPa) ranges, respectively ( Figure S2, Supporting Information). This suggests that the most enriched pathways in each stiffness comparison are largely driven by a subset of stiffness‐sensitive transcripts among the top differentially expressed genes.

To further characterize the enriched transcriptional processes, we performed overrepresentation analysis (ORA) using Gene Ontology biological processes (GO‐BP) database. Again, biological processes related to histone modifications, chromatin remodeling and epigenetics were significantly enriched in the (35 vs 180 kPa) stiffness comparison, occ seven out of the top 10 overrepresented GO‐BP terms (Figure 2G). Consistent with the pathway analysis, a wider range of GO terms were overrepresented in the (2 vs 180 kPa) pair, with cell adhesion and migration, angiogenesis and synapse assembly terms, among others, occupying the top 10 most enriched terms (Figure 2H).

Next, we sought to computationally deduce the upstream regulators that are likely responsible for the observed transcriptional programs or display an altered activity in response to the underlying substrate stiffness. We used a bioinformatic approach based on the *Expression2Kinase* (X2K) algorithm, which relies on the global pattern of transcriptome changes to construct a protein–protein interaction network in order to infer potential upstream regulators.^[^ ^16^ ^]^ We performed a transcription factor (TF) enrichment analysis on each of the three pairwise comparisons (Figure 2C,I–K).

In the soft‐to‐intermediate stiffness comparison (2 vs 35 kPa), we noted significant enrichment of transcriptional regulators related to endodermal/mesodermal differentiation (FOXA1, FOXA2, and SMAD4) and self‐renewal and stemness (NANOG and SALL4) (Figure 2I). In contrast, intermediate‐to‐rigid (35 vs 180 kPa) and soft‐to‐rigid (2 vs 180 kPa) comparisons were consistently enriched in TFs members of polycomb repressive complex 2 (SUZ12 and EZH2) which are known to function as heterochromatin modifiers.^[^ ^17^ ^]^ Intriguingly, the intermediate‐to‐rigid (35 vs 180 kPa) range uniquely showed enrichment of TFs related inflammation and cytokines signaling (STAT3 and IRF1) and adipogenic differentiation (PPARD and CEBPD) (Figure 2J,K). To further characterize the activity of upstream regulatory motifs, we performed complementary activity analysis using ISMARA (Integrated System for Motif Activity Response), which estimates TFs and miRNA activities at the proximal promoters sites using the whole transcriptome‐wide RNA‐seq data.^[^ ^18^ ^]^ ISMARA inferred activities across the mechanovariant substrates revealed that five of the top 15 motifs with the highest Z‐scores (TEAD3, TCF4, KLF4, SMAD3, HOXB2) are TFs known to be mechanoresponsive or respond to changes in matrix stiffness (Figure 2L).^[^ ^19^ ^]^ By cross‐examining the inferred TFs in X2K and ISMARA datasets, we found that majority of X2K predictions show consistent differential motif activity in ISMARA outputs (Figure S7, Supporting Information). We also examined the gene expression level of some of the inferred TF by rt‐qPCR, which showed no changes or correlation between the transcript levels and the computationally predicted activity (Figure S8, Supporting Information).

In the next step, we extended our analysis and used the DEGs and predicted TFs as seed inputs for the kinase enrichment analysis (KEA) module of the X2K tool. This analysis showed that mitogen‐activated protein (MAPK) kinases (ERK1/MAPK3, ERK2/MAPK1, and p38*α*/MAPK14) and the serine/threonine‐protein kinase (AKT1 and GSK‐3*β*) ranked among the top enriched kinases across the different pairwise comparisons (Figure 2M,N); and all of which have been implicated in mechano‐signaling in the context of stromal connective tissues.^[^ ^20^ ^]^ Experimental validation with immunoblotting confirmed that AKT1 and GSK‐3*β* phosphorylation are increased and repressed, respectively, with the increase in matrix stiffness (Figure 2P,Q and Figure S3, Supporting Information). Furthermore, we recently demonstrated that ERK1/2 acts a signaling checkpoint mediating the loss of mechanical tension in tendon explants.^[^ ^21^ ^]^ In addition, the X2K algorithm predicted distinct kinases that were enriched in a stiffness‐specific manner. While Janus kinase (JAK) family members (JAK1, JAK2, JAK3, and TYK2) were enriched in the intermediate‐to‐rigid (35 vs 180 kPa) stiffness range, Cyclin‐dependent kinases (CDK1, CDK2, CDK4, and CDK6), Casein kinases, and HIPK kinases (HIPK1 and HIPK2) subfamilies were substantially more enriched in the soft‐to‐rigid (2 vs 180 kPa) stiffness comparison (Figure 2M–O and Figures S2–S6, Supporting Information). This suggests that matrix stiffness potentially alters tendon stromal cells signaling through convergent (stiffness‐sensitive) and distinct (i.e., stiffness‐specific) pathways.

### 2.3 Global Transcriptomic Characteristics of Tenocytes Phenotypic Drift in TCP

Prolonged culture expansion on conventional tissue culture plastic (TCP) activates pro‐fibrogenic transcriptional and epigenetic programs that are imprinted as “mechanical memory” in stromal cells.^[^ ^8^, ^22^ ^]^ In addition, it has been reported that human tendon‐derived stromal fibroblasts undergo phenotypic drift when explanted and progressively passaged in vitro in the ultra stiff TCP.^[^ ^23^ ^]^ To assess the full extent of early transcriptomic responses of the TCP‐induced phenotypic drift, we initiated tendon stromal fibroblasts cultures from freshly isolated cells on conventional culture vessels without ever passaging the cells. When cells reached 70–80% confluency (approximately after 5–7 d), we collected the cells for bulk RNA sequencing and compared genome‐wide mRNA transcription to native, tissue‐level controls.

**Figure 3:**
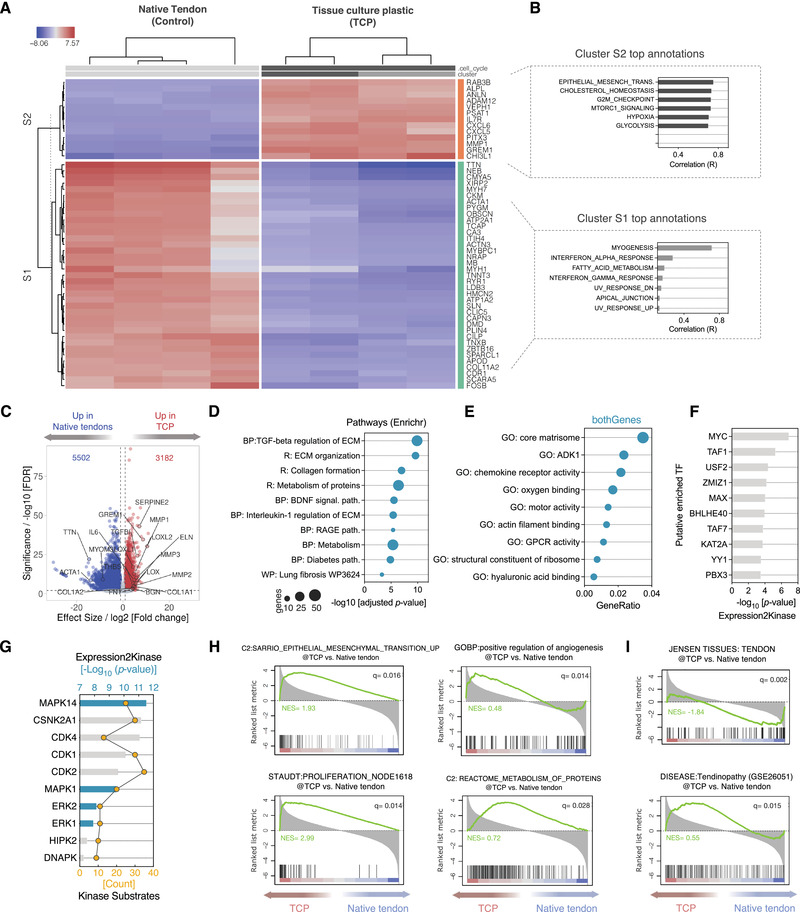
Global transcriptomic characteristics of tenocytes phenotypic drift on TCP. A) Heatmap of gene‐level hierarchical clustering of the top 50 differentially expressed genes (DEG) in tendon stromal fibroblasts cultured on tissue culture plastic (TCP) versus vehicle control. Columns represent individual samples (N = 4 biological replicates from different donors). Blue denotes downregulated genes; red denotes upregulated genes. B) Functional annotation of S1 and S2 gene clusters in the heatmap (encompassing top 500 DEGs). Bar plots depict the fisher‐weighted, average correlations of the cluster with the annotation terms queried against MSigDB Hallmark database. C) RNA‐seq volcano plot of DEGs of TCP‐cultured human tenocytes relative to native tendons. Colored dots show the 8684 significantly expressed genes, as determined by *DESeq2* methods, with the horizontal line corresponding to an FDR ≤0.01 and vertical lines are at a cutoff of log2[Fold change] ± 1. D) Enriched pathways analysis of a subset of DEGs using *Enrichr* queried against BioPlanet 2019 (BP), Reactome (R), WikiPathways 2019 (WP), and KEGG 2019 (K) Human databases. All hits had an adjusted *p*‐value < 0.05. E) Overrepresentation analysis (ORA) of Molecular Functions GO terms of upregulated and downregulated DEGs. Analysis was performed using a hypergeometric overrepresentation test against the GO database, with significance achieved at *q‐*value < 0.05. F) Bar plot depicts the predicted top 10 most significantly enriched transcription factors upstream of the DEGs. Predicted TFs are sorted by significance level (adjusted *p*‐value < 0.05). G) Top 10 kinases upstream of predicted TFs in (F) were identified using the Kinase Enrichment Analysis module of the *Expression2Kinase* pipeline. H) GSEA enrichment plots of some of the top differentially expressed gene sets. The plot's black vertical lines represent the ranked gene hits in the gene set. The green line represents the normalized enrichment score (NES). The more the NES curve is skewed to the upper left of the plot, the more the gene set is enriched in the TCP group. In contrast, when the curve shifts to the lower right, this reflects a more enrichment in the Native Tendon control group. The red‐to‐blue colored scale at the bottom represents the degree of correlation of genes in the TCP group with the phenotype in the gene set (Red: positive correlation; blue: negative correlation) or vice versa for the Native Tendon control group. Note the significant regulation of gene set terms related to EMT, cellular proliferation, metabolism, and angiogenesis. I) GSEA enrichment plots of tendon‐specific (Top) and tendinopathy (bottom) signatures.

Differential expression analysis revealed strong and significant regulation of 8684 DEGs between TCP cultures and native tendons, among which 5502 were upregulated and 3182 were downregulated at a threshold of |fold change (FC)| ± 1 and adjusted *p* < 0.01 (**Figure** **3**A–C and Figure S9, Supporting Information). Notably, differentially upregulated genes in TCP substrates showed positive correlation with reference database MSigDB Hallmarks gene sets: epithelial‐mesenchymal transition (EMT), hypoxia, and metabolism [represented by cholesterol homeostasis, MTORC1 signaling, and glycolysis] (Figure 3B). In contrast, myogenesis gene set showed the strongest correlation (Pearson *r* = 0.81) with the upregulated genes in the Native tendon contrast, possibly reflecting an overlap between tendon and muscle transcriptomes.

To systematically characterize the enriched transcriptional programs, we performed pathway enrichment analysis on DEGs of S2 subcluster using the tool EnricrR.^[^ ^15a,b^ ^]^ Approximately half of the top 10 enriched hits were pathways related to fibroinflammatory regulation of the ECM, while 4/10 of the enriched terms are representative of metabolic pathways (Figure 3D). We then carried out an over‐representation analysis (ORA) to examine the gene ontology (GO) terms that are over‐represented in all the up‐regulated and down‐regulated DEGs.^[^ ^24^ ^]^ GO terms related core matrisome topped the overrepresented gene sets, with consistent enrichment of terms related to chemokine activity, cytoskeletal remodeling, and GPCR signaling (Figure 3E).

Next, we delineated the regulatory signaling kinases and transcriptional factors upstream of the DEGs. X2K TF module identified 32 TFs that may potentially regulate the DEGs (Figure 3F). The top 10 TFs were dominated by transcriptional regulators known to be involved in cell cycle progression, apoptosis, or cellular differentiation (MYC, MAX, BHLHE40) or chromatin remodeling (KAT2A and YY1). Further, X2K kinase enrichment analysis predicted a total of 98 significant kinases (Hypergeometric *p*‐value of < 0.05), of which the top 10 most significant kinases are dominated by mitogen‐activated protein kinases (MAPK14, ERK1, ERK2) and cyclin‐dependent kinases (CDK1, CDK2, CDK4) (Figure 3G and Figure S10, Supporting Information). Finally, to get a global unbiased insight on the potential phenotypes associated with these altered gene programs, we carried out preranked gene set enrichment analysis (GSEA).^[^ ^25^ ^]^ We found that epithelial‐mesenchymal transition (NES 1.93), proliferation (NES 2.99), regulation of angiogenesis (NES 0.48) and metabolism of proteins (NES 0.72) gene sets are among the most significant signatures with positive correlation to TCP cultures (Figure 3H). Interestingly, TCP transcriptome showed significant enrichment in tendon‐related signatures, with negative correlation with Jensen TISSUES (Tendon) and positive correlation with DISEASE: Tendinopathy signatures (Figure 3I).

Collectively, these data provide strong evidence that culture expansion in conventional TCP polystyrene vessels contributes to fibro‐inflammatory and metabolic activations of tendon‐derived fibroblasts, with the divergence of the whole transcriptome towards a pathological signature closely resembling that of pathogenic tendinopathic tendons.

### 2.4 Tenogenic Mechanovariant Substrates Abrogate TCP‐Mediated Inflammatory Programs and Favor Tendon‐Specific Expression Signature

**Figure 4:**
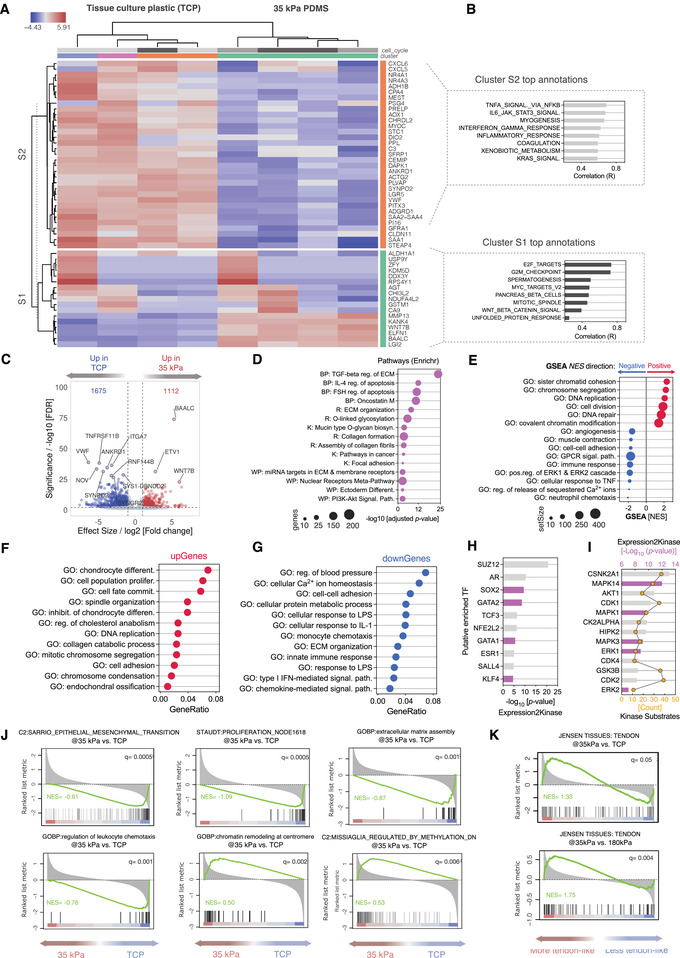
Tenogenic mechano‐variant substrates abrogate TCP‐mediated inflammatory programs and favor tendon‐specific expression signature. A) Heatmap of gene‐level hierarchical clustering of the top 50 differentially expressed genes (DEG) in tendon stromal fibroblasts cultured on tenogenic substrates (*E*. ≈35 kPa) versus TCP control. Columns represent individual samples (*N* = 4 biological replicates from different donors). Blue denotes downregulated genes; red denotes upregulated genes. B) Functional annotation of S1 and S2 gene clusters in the heatmap (encompassing top 500 DEGs). Bar plots depict the fisher‐weighted, average correlations of the cluster with the annotation terms queried against MSigDB Hallmark database. C) RNA‐seq volcano plot of DEGs of 35kPa‐cultured human tenocytes relative to TCP controls. Colored dots show the 2787 significantly expressed genes, as determined by *DESeq2* method, with the horizontal line corresponding to an FDR ≤0.01 and vertical lines are at a cutoff of log2[Fold change] ± 1. D) Enriched pathways analysis of a subset of DEGs using *Enrichr* queried against BioPlanet 2019 (BP), Reactome (R), WikiPathways 2019 (WP), and KEGG 2019 (K) Human databases. All hits had an adjusted *p*‐value < 0.05. E) Pre‐ranked Gene Set Enrichment Analysis (GSEA) of positively and negatively enriched cellular components by Normalized Enrichment Score (NES) in 35 kPa cultured cells (Adjusted *p*‐value < 0.05). F,G) Overrepresentation analysis (ORA) of Molecular Functions GO terms of F) upregulated and G) downregulated DEGs. Analysis was performed using a hypergeometric over‐representation test against the GO database, with significance achieved at *q‐*value < 0.05. H) Bar plot depicts the predicted top 10 most significantly enriched transcription factors upstream of the DEGs. Predicted TFs are sorted by significance level (adjusted *p*‐value < 0.05). I) Top 10 kinases upstream of predicted TFs in (F) were identified using the Kinase Enrichment Analysis module of the *Expression2Kinase* pipeline. J) GSEA enrichment plots of some of the top differentially expressed gene sets. The Plot's black vertical lines represent the ranked gene hits in the gene set. The green line represents the normalized enrichment score (NES). The more the NES curve is skewed to the upper left of the plot, the more the gene set is enriched in the 35 kPa group. In contrast, when the curve shifts to the lower right, this reflects a more enrichment in the TCP control group. The red‐to‐blue colored scale at the bottom represents the degree of correlation of genes in the 35 kPa group with the phenotype in the gene set (Red: positive correlation; blue: negative correlation) or vice versa for the TCP control group. Note the significant negative regulation of gene set terms related to EMT, cellular proliferation, ECM assembly, and inflammation. K) GSEA enrichment plots of tendon‐specific (Top) and tendinopathy (bottom) signatures.

Next, we sought to assess how mechanovariant silicone substrates modulate early transcriptional responses of tendon stromal cells in comparison to ultra‐stiff TCP. We compared transcriptome profiles of soft, intermediate, and rigid‐cultured tendon cells to conventional TCP surfaces (**Figure** **4** and Figures S13 and S14, Supporting Information). Here, we will only highlight the results from the intermediate (35 kPa) stiffness in the interest of maintaining the focus of the discussion on tendon‐related responses.

When cells were conditioned to tenogenic PDMS substrates (*E*. 35 kPa), unsupervised genes subclusters showed consistent and negative correlations with Hallmark gene set annotations related to inflammation (e.g., TNFA Signaling via NFKB, IL6 JAK STAT3, Inflammatory Response) and Myogenesis in the MSigDB Hallmark database (Figure 4A,B). Differential analysis revealed 2787 significantly expressed genes with 1112 upregulated and 1675 downregulated genes at a threshold of |fold change (FC)| ± 1 and adjusted *p* < 0.01 (Figure 4C). Using *Enrichr*, pathway analysis uncovered the dominance of fibro‐inflammatory related pathways such as TGF‐*β*1 regulation of ECM, collagen formation, IL‐4 regulation of apoptosis and ECM organization (Figure 4D). Genes overlapping with PI3K‐Akt signaling pathway were also significantly enriched, in consistence with the comparison across the PDMS stiffnesses (Figure 2F). Preranked GSEA by *fgsea* showed analogous enrichment of GO terms with positive regulation of chromatin remodeling and DNA replication and repair processed (Figure 4E). Interestingly, the enrichment of gene sets related to angiogenesis, ERK1/2 cascades and inflammatory pathways in silicone substrates showed consistent negative gene regulation across all PDMS stiffnesses in comparison to TCP (Figure 4E and  Figures S12E and S13E, Supporting Information).

This hints at the possibility that PDMS substrates tend to abrogate the strong TCP‐mediated pro‐inflammatory and stress responses. Furthermore, ORA analysis showed significant over‐representation of GO terms associated with cell fate commitment and musculoskeletal cells differentiation [represented by chondrocyte differentiation, inhibit. of chondrocyte differentiation and endochondral ossification] in the upregulated genes (Figure 4F). In contrast, more diverse GO processes were over‐represented in the down‐regulated genes dominated by inflammation (seven GOs), in addition to protein metabolism, ECM organization and calcium ion homeostasis (Figure 4G).

Reconstruction of upstream TFs and regulatory kinases deduced consistent enrichment of TFs known to be involved in self‐renewal and stemness [SOX2, GATA2, GATA1, KLF4] across all PDMS variations relative to TCP. This implies that mechanovariant substrates may enrich or condition a subpopulation of tendon cells towards a more progenitor/stemness‐like state (Figure 4H and Figures S13H and S14H, Supporting Information).^[^ ^26^ ^]^ In addition, SUZ12 was the top deduced TF across all the PDMS comparisons, while ERK1/2, GSK‐3 and AKT1 kinases over‐populated the list of predicted kinases (Figures 4H,I and 2P,Q, Figures S3, S13, and S14, Supporting Information). Surprisingly, 35 kPa tenogenic substrates induced expression patterns that positively correlated with signatures suggestive of epigenetic processes (chromatic remodeling: NES 0.50, Methylation regulation: NES 0.53) (Figure 4J), while strongly correlated with tendon tissue‐specific transcriptomic signature (NES 1.33) (Figure 4K). This was evident when we compared the mRNA expression profiles of cells cultured on 35 kPa substrates to freshly extracted tendon stromal cells and TCP cultures. Transcripts for markers of tendon fibroblasts (*TNMD*, *SPP1*), and progenitor differentiation (*ALPL*, *PPARG*) showed expression trends that were closer to those of freshly isolated cells than to TCP controls (Figure S11, Supporting Information). More importantly, tendon stromal cells only maintained the expression of *TNMD* transcripts at passage 2 if cultures were initiated in 35 kPa substrates, or if they were initiated in TCP and then transferred to soft (2 kPa) or intermediate (35 kPa) stiffnesses, but not to the rigid 180 kPa substrates (Figure S12, Supporting Information). Together, this suggests that the 35 kPa stiffness range favors maintaining a tendon‐specific transcriptional program in tendon stromal fibroblasts.

## 3 Discussion

In this work, we have established a user‐friendly, 2D mechanovariant cell culture system using silicone elastomer gels which is amenable to large‐scale cell expansion practices. We have used this new biomaterial system to profile early stiffness‐sensitive transcriptional mechano‐responses of human tendon‐derived stromal cells and defined a stiffness range (*E*. modulus ≈35 kPa) that reinstates a tendon‐like transcriptomic signature in tendon stromal cells in vitro.

A major motivation behind this work lays in overcoming the long‐standing technical challenges in using tunable mechanovariant substrates in routine cell culture practice. We addressed this issue by using commercially available, pure polydimethylsiloxanes silicone components that are devoid of silica nanoparticle fillers.^[^ ^27^ ^]^ We formulated the constituent precursors to yield a balanced two‐part liquid components that can be mixed at a (1:1) mixing ratio, irrespective of the target stiffness. With an important step that dramatically simplifies later surface preparation, we copolymerized undecenoic acid in the reaction mix to introduce carboxylic acid groups that enabled direct covalent coupling of primary amines using the EDC/NHS crosslinking chemistry. Thus, we circumvented the limitations of using the unstable, UV light‐dependent sulfo‐SANPAH heterobifunctional crosslinking chemistries. Furthermore, we eliminated the need for using plasma surface activation methods which are known to alter PDMS mechanical properties by introducing a thin, stiff plasma‐oxidized layer.^[^ ^28^ ^]^ This allowed us to produce large‐scale mechano‐variant substrates inside sealed tissue culture flasks under sterile conditions. More importantly, we were able to use the platform to propagate freshly isolated tendon fibroblasts at all stiffness ranges (from 2 to180 kPa) without ever exposing the cells to the potentially confounding effects of TCP‐mediated mechanical dosing.^[^ ^22^, ^29^ ^]^

Perhaps one of the most striking findings of this study is that the intermediate range of stiffness (≈35 kPa) conditioned tendon‐derived stromal cells transcriptome towards a tendon‐specific tissue signature.

Substrate stiffness‐driven tenogenic differentiation of bone marrow stromal cells has been described for many years. Our previous work has established for the first time that intermediate matrix stiffnesses (20–40 kPa) and collagen type I ECM ligands direct the differentiation of bone marrow mesenchymal stromal cells towards tendon lineage specification.^[^ ^13^, ^14^ ^]^ Similarly, Rehmann and co‐workers showed that synthetic poly(ethylene glycol)‐based elastic substrates promoted the expression of tendon lineage‐associated genes in human mesenchymal stem cells, with the 50 kPa modulus being the optimal stiffness.^[^ ^13b^ ^]^ Our results here extend these observations to tendon‐derived stromal fibroblasts, and suggest that resident fibroblasts sense the optimal tendon moduli within a narrow range of values approximating that of in vivo cell‐scale microenvironmental stiffness. Tendon tissue micro‐ and nanoscale indentation modulus has been characterized, and indeed it has been reported that cell‐scale elastic modulus spans a range of tens of kPa, depending on the methods used as well as the anatomical source of the tendon.^[^ ^30^ ^]^

These findings are consistent with the current view from other biological systems that the optimal mechanical microenvironment for a given cellular behavior in vitro often correlates with the corresponding elasticity of the living tissue in vivo.^[^ ^31^ ^]^ In muscle, the optimal substrate stiffness that facilitates myotube differentiation and striation matches the elasticity of healthy muscle at *E*. ≈12 kPa.^[^ ^32^ ^]^ Gilbert et al. went a step further and showed that muscle stem cells propagated in physiologically relevant, muscle‐like rigidities (12 kPa) did not only maintained self‐renewal capacity in vitro, but also exhibited improved muscle engraftment and functional regeneration in mice in vivo.^[^ ^33^ ^]^ Similarly, oligodendrocyte neuro‐progenitor cells lose their normal function as their micro‐niche stiffens with aging, yet they can be functionally rejuvenated when transplanted to a compliant synthetic niche with stiffnesses that mimic the elasticity of young brains.^[^ ^34^ ^]^

In lungs, compliant healthy lung‐like tissue stiffness completely suppresses collagen type I secretion and proliferation of pro‐fibrotic, activated fibroblasts.^[^ ^3^ ^]^ Collectively, these intriguing observations indicate that stromal cells are inherently sensitive to matrix stiffness. Furthermore, it implies that there might be unifying biological mechanisms that facilitate cells sensing of optimal tissue‐specific stiffness cues, possibly through (epi)‐genetically imprinted “homeostatic set‐points.”^[^ ^4^, ^35^ ^]^

Our transcriptomic data hints that such regulatory (epi‐)genetic mechanisms might be implicated in defining a tensional “homeostatic set‐point” in tendon cells. Soft (2 kPa) and rigid (180 kPa) stiffnesses model states of low and elevated matrix tension, respectively. Pairwise comparison of DEGs revealed that stiffness deviations from the tendon‐like elasticity of *E*. ≈35 kPa correlated with expression of histone‐related genes (H2A, H2B, H3, and H4 family members) (Figure 3B). Although such differential expression may reflect differences in replicative cell‐cycle progression, several lines of evidence argue against that. First, bioinformatics analysis showed that mechanically regulated pathways, i.e., histone modifications and Hippo pathway, were the top two most enriched pathways in the DEGs of intermediate stiffness (35 kPa) compared to the stiffer substrates (180 kPa). Second, 70% of the top overrepresented GO terms are related to epigenetic processes that are reported to be involved in stiffness‐sensing and mechanical memory (Figure 2D,G). Biophysical cues, including stiffness, can regulate cells’ “epigenetic states” by altering chromatin organization and accessibility.^[^ ^36^ ^]^ Human mesenchymal stromal cells cultured on soft or stiff microenvironments showed differential responses in histone acetylation and chromatin organization that were dependent on the stiffness cues and the length of exposure to stiff hydrogels.^[^ ^22^ ^]^ Along the same line, matrix stiffening is implicated in the pathological activation of cardiac stromal cells through a process involving histone deacetylases and chromatin remodeling.^[^ ^22^ ^]^

In the context of tendon, Heo and co‐workers have recently demonstrated that substrate stiffness alters chromatin spatial organization (Histone‐H2B nanodomains) of tendon‐derived stromal cells, which is in agreement with our observations.^[^ ^37^ ^]^ When tendon fibroblasts were conditioned to soft (3 kPa) methacrylated hyaluronic acid hydrogels or ultra‐stiff glass surfaces, histone‐H2B nanodomains condensed and relocated to the nuclear periphery. This spatial chromatin condensation was reminiscent to what they observed in cells obtained from diseased tendinopathic or aged tendons. Strikingly, tenocytes that were cultured on substrates with tendon‐like stiffness (30 kPa) exhibited diffuse chromatin distribution throughout the nucleus akin to the spatial patterning observed in cells derived from healthy young tendons.^[^ ^38^ ^]^ While our data does not establish direct causal relationship between substrate stiffness and chromatin architecture, upstream TFs analysis predicted enrichment of several transcriptional effectors known to be involved in epigenetic machineries. Notably, SUZ12 is the only inferred TF that is shared across all the stiffness comparisons (Figure 2I–K and Figure S7, Supporting Information). In the most rigid condition (180 kPa), SUZ12 moves up the list to be the most significantly enriched putative TFs while EZH2 appears among the top 10 most enriched TFs. Both SUZ12 and EZH2 form the core subunits of the polycomb repressive complex 2 (PRC2). PRC2 complex binds remodeled heterochromatin and catalyzes the formation of H3K27me3 epigenetic mark, which is widely acknowledged to play a key role by acting as a “mechanical rheostat” in response to dynamic mechanical loading or in instilling epigenetic mechanical memory in mesenchymal stromal cells.^[^ ^36^, ^39^ ^]^

Another key insight from this work is that we documented the extent of the transcriptome‐wide phenotypic drift of freshly isolated tendon stromal cells when maintained in conventional tissue culture plastic (TCP). Progressive cell expansion in ultra‐stiff TCP is widely acknowledged as a key driver of sustained activation of tenocytes towards pro‐fibrotic myofibroblasts.

Our data here show that standard TCP‐culture significantly alters the native phenotype of tendon cells, even without ever expanding these cells in tissue plastic (i.e., initiating and collecting the cells for downstream analysis at passage 0) (Figure 3 and Figure S9, Supporting Information). These observations are in agreement with recent findings made by our group and others.^[^ ^23^, ^40^ ^]^ van Vijven et al. has shown that expansion of mouse tenocytes in 2D TCP substrates induced loss of native phenotype of mouse tendon‐derived cells. Consistently, Imada and co‐workers reported similar findings and implicated serum supplementation in culture medium in mediating osteoblastic‐like changes in rat Achilles tenocytes. Interestingly, while the tenogenic markers were strongly suppressed in our TCP conditions, the osteogenic marker *ALPL* (Alkaline phosphatase) was significantly upregulated compared to native tendons in our data (Figure S9A,B, Supporting Information). One intriguing question is whether this differential expression reflects an underlying differentiation of subpopulation of tendon‐resident stem cells or a serum‐dependent proliferation ALPL^+^ cells.

Furthermore, the detailed transcriptome analysis revealed that tendon cells in stiff TCP cultures exhibited strong transcriptional signatures suggestive of angiogenesis, fibro‐inflammatory (EMT, TGF‐*β*1 pathway), and metabolic (glycolysis) activation. Two independent reports have established that TCP per se activates quiescent stromal cells into myofibroblast‐like phenotype, through coordinated short‐term signaling cascades and long‐term epigenetic mechanisms.^[^ ^8^, ^29^ ^]^ PI3k/AKT pathway was implicated in mediating the short‐term effects of pathologically‐stiff matrix, while we recently uncovered that ERK1/2 kinases are central checkpoints relaying short‐term signaling responses to mechanical stress in tendons.^[^ ^21^, ^29^, ^41^ ^]^ ERK1/2 were overrepresented across all stiffness comparison in this work, further reinforcing its role in matrix‐mediated mechano‐responses.^[^ ^21^ ^]^

Additionally, stiff matrix mechanical cues were shown to rewire the metabolic activity of cells in vitro toward “mechanoresponsive glycolysis,” through a mechanism involving cytoskeletal tension, actin organization, and proteasomal degradation of the metabolic enzyme phosphofructokinase (PFK).^[^ ^42^ ^]^ Moreover, increased glucose metabolism, through glycolysis, was reported to modulate the transcriptional activity of YAP/TAZ suggesting that mechanoresponsive glycolysis may instill a self‐amplifying feed‐forward loop between substrate stiffness and mechanosignaling.^[^ ^43^ ^]^ Interestingly, our PDMS mechano‐variant substrates completely abrogated the angiogenesis, fibro‐inflammatory and metabolic signatures when compared to TCP transcriptome (Figures 3 and 4 and Figures S13 and S14, Supporting Information). Collectively, this suggests that PDMS elastomeric gels not only provide an optimal “tenogenic” stiffness but also mitigate the undesirable side effects of conventional tissue culture plastics.

In summary, we describe a novel PDMS‐based culture platform that greatly improves the scalability, reproducibility, and ease of handling of mechano‐variant substrates. We determined that 35 kPa substrates tend to support tendon marker expression while mitigating activation of pathways associated with loss of tensional homeostasis. Finally, we highlight that conventional TCP culture of tendon cells activates proinflammatory, proangiogenic and metabolic pathways that markedly differ from freshly isolated tendon cells and which brings the scientific probity of their use into question.

## 4 Experimental Section

### Preparation of Silicone Mechano‐Variant Substrates

Silicone gels were prepared by mixing pure liquid vinyl‐terminated polydimethylsiloxane (PDMS) (1000 cSt – DMS‐VM31, Lot. 1K‐39727. Gelest), hydride‐terminated PDMS (500 cSt—DMS‐HM25, Lot. 5L‐26253. Gelest), 7–8% (Methylhydrosiloxane)‐dimethylsiloxane copolymer, trimethylsiloxy‐terminated (AB146377, abcr GmbH) and Platinum‐divinyltetramethyldisiloxane catalyst (AB146697, abcr GmbH). A 0.2% (v/v) of 10‐Undecenoic acid (124672, Sigma‐Aldrich) was added to the mix to facilitate direct coupling of primary amines. The gels’ Young moduli were tuned by varying the amounts of crosslinker, vinyl‐terminated, and hydride‐terminated PDMS in the mix, while keeping the ratio of the platinum catalyst constant at 0.015% (v/v). To polymerize the gels, the silicone solution was thoroughly mixed, degassed under vacuum for 30 min and allowed to set at room temperature (for a total of 1 h). Silicone mixture was then poured into desired cell culture vessels and cured at 70 °C for another 23 h. Cured PDMS substrates were sterilized using UV light and 70–80% ethanol under aseptic conditions.

### Material Stiffness Measurements

PDMS substrates were prepared in 40 mm petridishes (2 g per dish). Force–displacement measurements were carried out using capacitive piezoresistive MEMS force sensor mounted on an XYZ micromanipulating arm in a vertical configuration (FT‐RS1002 Microrobotic System: FemtoTools AG, Switzerland). Micointendation measurements were collected using a sensor with glass sphere mounted on its tip (sphere diameter ≈232 µm). Before indentation, indenting spheres were lubricated with silicone oil by carefully driving it onto an oil‐saturated piece of soft PDMS‐elastomer. Excess oil material was stamped on a dry silicone rubber surface. Measurements were collected in Step mode with 30 steps s^‐1^ and 30% steps size (≈0.2 µm), i.e., 6 µm s^−1^. Hertz model “sphere indenting elastic half‐space” was used to determine the *E. modulus*.

### PDMS Functionalization and ECM Coating

To facilitate cell adhesion, PDMS substrates were functionalized with in‐house prepared rat tail collagen type I using the mild Sulfo‐NHS plus EDC (carbodiimide) crosslinking procedure (carboxyl‐to‐amine). Substrates were sterilized with 80% ethanol (v/v), then washed three times with MilliQ water. PDMS surfaces were activated with Sulfo‐NHS/EDC: (3 mg) EDC plus (3 mg) NHS in (1 mL) sterile MES buffer (2‐(N‐morpholino)ethanesulfonic acid) buffer (28390, Thermo Scientific) for 20–30 min at RT. Collagen type I was diluted to 200 µg mL^−1^ in ice cold PBS, and used to coat the PDMS surface overnight at 4 °C (at a ligand density of 20 µg cm^−2^).

### Isolation of Human Tendon‐Derived Stromal Cells

Human tendon biopsies were obtained from healthy donors undergoing autograft tendon transfer procedures for the surgical repair of anterior cruciate ligaments.^[^ ^44^ ^]^ Excess samples that would have otherwise been discarded were collected with informed donor consent in compliance with the requirements of the Declaration of Helsinki, the Swiss Federal Human Research Act (HRA), and Zurich Cantonal Ethics Commission (Approval number: 2015‐0089, 2020‐01119). Immediately after surgical dissection, tendon samples were placed in (DMEM)/F12 medium (D8437, Sigma‐Aldrich) in the operating room and were placed in 4 °C. In the cell culture lab, samples were thoroughly washed in PBS to remove blood or attached muscle debris. Tendon tissue was cut into small pieces (approximately 1 mm^3^ cubes). Human tendon‐derived stroma cells were isolated by collagenase digestion using collagenase, Type I (17018029, Gibco) at 37 °C for 6–12 h digestion.^[^ ^45^ ^]^ After digestion, freshly isolated single cells were seeded directly onto PDMS substrates without further expansion on tissue culture flasks (i.e., passage 0). During the experiments, cells were maintained in Dulbecco's modified Eagle's medium with L‐glutamine, 15 × 10^‐3^ m HEPES and sodium bicarbonate (DMEM/F12–D8437, Sigma‐Aldrich), supplemented with 10% heat‐inactivated fetal bovine serum (FBS – 10 500, Gibco), 1% (v/v) penicillin–streptomycin (P/S, P0781, Sigma‐Aldrich), 1% (v/v) Amphotericin B (15290018, Gibco). Cells were maintained in a culture incubator set at 37 °C and 5% CO_2_, and fresh media were replenished every 3–4 d until cells reached 70–80% confluency. Cells were counted manually using hemocytometer to calculate population doubling levels (PDL) and population doubling times (PDT).

### RNA Extraction: Cells in PDMS Substrates

Cells were washed with PBS and were lysed in situ using Qiagen RLT Plus lysis buffer (1053393, Qiagen). Cell lysates were snap‐frozen in liquid nitrogen and stored at ‐80 °C until further use. To extract the RNA, samples were thawed on ice, vortexed for 1 min, and homogenization using QIA shredder columns (79654, Qiagen). Total RNA was isolated using RNeasy Plus Micro Kit (74034, Qiagen), including a genomic DNA removal step with Qiagen gDNA Eliminator spin columns and on‐column DNase digestion (12185010, Invitrogen). RNA concentration was measured using Qubit RNA HS Assay Kit (Q32852, Invitrogen).

### Human Tendon Tissues

Control tissues were freshly snap‐frozen in liquid nitrogen immediately after the surgical operation. On the day of RNA extraction, samples were transferred to a supercooled Spex microvial grinding cylinder (6757C3, SPEX SamplePrep) containing 100 µL of GENEzol reagent (4402‐GZR200 Labforce LSBio). Samples were pulverized in a bath of liquid nitrogen using a cryogenic mill (Spex6775 FreezerMill) for 2–4 milling cycles at 15 cps. The resulting pulverized tissue lysate was further solubilized in an additional (1–1.5) mL GENEzol. Insoluble ECM fragments were disrupted by passing the whole lysate approximately 10 times through a 21G syringe needle, followed by centrifugation at 5000 g for 5 min.

To extract the RNA, cleared homogenate was thawed and thoroughly mixed with (200 µL) of chloroform (102445, Merck) at a mixing ratio of 1:5. After centrifugation (12 000 g, 15 min, 4 °C), the RNA containing aqueous phase was carefully pipetted, mixed with an equal volume of 70% EtOH. Further RNA clean‐up was carried out using Mini Scale Kit for human tissue (Invitrogen, 12183018), including an on‐column DNase digestion (12185010, Invitrogen), according to the manufacturer's instructions. RNA amounts were quantified with Qubit RNA HS Assay before submitting the samples for RNA‐sequencing.

### Genome‐Wide RNA Sequencing (RNA‐Seq)

RNA‐Seq libraries construction and sequencing were carried out by GENEWIZ (Leipzig, Germany). Briefly, mRNA integrity and yield were assessed with Agilent Fragment Analyzer (Agilent Technologies, USA). For mechano‐variant culture samples, all the samples passed the quality control criteria for sequencing with an RNA quality number (RQN) ≥ 9. RNA‐Seq libraries were prepared with poly‐A selection enrichment using NEBNext Ultra II Directional RNA Library Prep Kit for Illumina, following manufacturer's instructions (NEB, Ipswich, MA, USA). For native human tendons, libraries were prepared using a ribosomal RNA depletion protocol using NEBNext rRNA Depletion Kit (Human/Mouse/Rat) and NEBNext Ultra II Directional RNA Library Prep Kit for Illumina (NEB, Ipswich, MA, USA). Libraries were sequenced on the Illumina NovaSeq 6000 instrument in Pair‐End (PE) configuration to a sequencing depth of at least 20 million reads.

### Data Preprocessing and Bioinformatics Analysis

Primary level bioinformatics analysis was carried out using the R package ezRun, which is implemented within the SUSHI framework of the Functional Genomics Center Zurich (ETH Zurich and the University of Zurich).^[^ ^46^ ^]^ Quality of the raw RNAseq data was verified with FastQC and MultiQC (version 1.9).^[^ ^47^ ^]^ Raw.fastq files were mapped to the human genome reference (Ensembl GRCh38.p10 – Release_91‐2018‐02‐26) with STAR aligner software.^[^ ^48^ ^]^ Reads counts per gene were calculated using featureCounts function against the reference genome (Ensembl version 78).^[^ ^49^ ^]^ Differential expression analysis was performed by using the *DESeq2* R package (version 4.0.3) using default parameters.^[^ ^50^ ^]^ Pathway enrichment analysis of differentially expressed genes or selected subclusters of genes of interest was carried out using *Enrichr* tool.^[^ ^15a,b^ ^]^ Gene lists were interrogated against KEGG, BioPlanet, Reactome and/or WikiPathways databases. Gene set functional enrichment was performed using two complementary methods: 1) pre‐ranked Gene Set Enrichment Analysis using *fgsea* [v1.16.0],^[^ ^51^ ^]^ 2) gene set overrepresentation analysis (ORA) implemented within clusterProfiler package [v3.18.0].^[^ ^24^ ^]^ For ORA analysis, *p*‐values were adjusted for multiple hypothesis testing using hypergeometric overrepresentation testing. Analyses were performed on both upregulated and down‐regulated genes, unless specified other in the figure legends. For tertiary functional analysis of expression signatures, raw read counts were imported into Omics Playground Suite (BigOmics Analytics, Switzerland) and implemented in RStudio (v. 1.3.1093).^[^ ^52^ ^]^ Functional annotations of heatmap clusters were defined based on geneset‐level, Fisher‐weighted correlation with the reference database Molecular Signatures Database (Hallmark collection, MSigDB).^[^ ^25^, ^53^ ^]^ Volcano plots were visualized using VolcaNoseR package.^[^ ^54^ ^]^

### Transcription Factors and Kinase Enrichment Analyses

Upstream regulators (kinases and transcription factors) that are likely responsible for the changes in gene expression were computationally deduced using *Expression2Kinases* toolkit (MacOS, Version 1.6.1207).^[^ ^15a^ ^]^ Top DEGs (up to 3000 genes) were fed into *Expression2Kinases* pipeline which applies enrichment algorithm to predict and rank potential transcription factors regulating the queried DEGs. Next, it constructs a protein–protein transcriptional regulatory subnetwork, which is seeded into the Kinase Enrichment Analysis (KEA) module.^[^ ^55^ ^]^ Predicted kinases were mapped to kinome tree dendrogram using Coral tool and phylogenetic information described elsewhere.^[^ ^56^ ^]^

### Transcription Factor and miRNA Activity Analysis

TF and miRNA activity analysis was conducted using ISMARA (Integrated System for Motif Activity Response Analysis) developed by Balwierz et al.^[^ ^18^ ^]^ ISMARA infers the regulatory motifs activity by computationally reconstructing the genome‐wide regulatory circuitry that drive the observed changes in gene expression. It identifies proximal promoters and quantifies their expression followed by fitting a linear model (Bayesian inference). Adaptor trimmed fastq.gz RNA‐seq files were uploaded to (https://ismara.unibas.ch/mara/) using the ISMARA python script, including a sample averaging step. To determine the direction of motif activity, the *Ζ*‐score of each motif was multiplied by the sign of Pearson correlation between the motif and its target genes, as described in ref. [57].

### Real‐Time Quantitative PCR (RT‐qPCR)

Real‐time quantitative PCR was carried out using CFX Opus 384 Real‐Time PCR System (12011452, Bio‐Rad) and TaqMan Assays. A total of 200 ng of RNA were reverse transcribed to cDNA using High‐Capacity RNA‐to‐cDNA Kits (4387406 or 4368814, Applied Biosystems). RT‐qPCR was performed with 2 µL cDNA and 8 µL of BioRad SSO Advanced Universal Probes Supermix (containing 5 µL Supermix, 0.5 µL of TaqMan primer, 2.5 µL of RNAse free ultrapure water) to a total volume of 10 µL (in technical duplicates). Relative expression was calculated using the comparative 2^ΔCT^ method.^[^ ^58^ ^]^ TaqMan primers assay IDs are listed in the online supplementary material 546 (Table S2, Supporting Information).

### Western Blotting

Cells were seeded on mechano‐variant substrates at a seeding density of 5000 cell cm^−2^ (in six‐well plates). After 24 h, cell lysates were collected by first washing the cells twice with ice cold PBS followed by addition of 100 µL per well of RIPA buffer (R0278, Sigma‐Aldrich) supplemented with Proteoloc Protease Inhibitor Cocktail EDTA‐FREE (EXP 42516, Lucerna Chem) and Phosphatase Inhibitor Cocktail I (P2850, Merck).

Cell lysates were incubated in ice for 5 min, and then collected with a scrapper. Samples were sonicated on ice for 90 s and then spun down. Total protein concentration was determined using Precision Red Advanced Protein Assay (Cytoskeleton, ADV02‐A). Next, samples were boiled at 94 °C for 5 min in Laemmli SDS sample buffer (15493939, Thermo Scientific). 4–15% Mini‐PROTEAN TGX Stain‐Free Protein Gels were used to resolve the cellular proteins (4568086, Bio‐Rad). SDS gels were then transferred to low fluorescence (LF) PVDF membrane using Trans‐Blot Turbo RTA Mini LF PVDF Transfer Kit, (1704274, Bio‐Rad). Subsequently, membranes were blocked with EveryBlot Blocking Buffer (Biorad, 12010020) for 5 min at room temperature. Membrane blots were incubated with primary antibodies diluted in 3% BSA in TBS‐Tween (wt/v) at 4 °C overnight. Next, membranes were washed three times with 2.5% milk in TBS‐Tween and incubated with secondary antibodies for 1 h at room temperature. After extensive washing, membranes were incubated with UltraScence Pico Ultra Western ECL Substrate (BHX‐CCH345, Lucerna Chem) and developed using Chemidoc MP Imaging System (Bio‐Rad). After probing the phosphorylated proteins, blots were stripped with Restore Plus Western Blot Stripping Buffer (46430, Thermo Scientific), blocked again, and reprobed with primary antibodies against the total protein of the same target. The Stain‐Free total protein signal was used as a loading control for normalization. List and dilutions of primary and secondary antibodies are summarized in (Table S3, Supporting Information).

### Imaging and Confocal Microscopy

Cells were seeded at low seeding density 150 cell cm^−2^ on PDMS coated glass‐bottom plates (P24‐1.5H‐N, Cellvis). After 24 h, cells were washed in PBS (with calcium and magnesium) (14040117, Gibco), then fixed with prewarmed 4% neutral buffered formaldehyde solution (ROTIHistofix 3105.2, Carl Roth) for 10 min at room temperature. Cells were permeabilized with 0.1% (v/v) Triton X‐100 (93418, Sigma) in PBS for 10 min at room temperature. After washing, cells were incubated with Phalloidin AlexFluor 488 (Invitrogen, A‐34055) and nuclei were counterstained with NucBlue. Fluorescence images were acquired with the iMic spinning disk confocal microscope, equipped with a Hamamatsu Flash 4.0 sCMOS camera and a SOLE‐6 Quad laser (Omicron), using 10× (N.A. 0.4) objective (Olympus UPLSAPO). Cell spreading area was quantified using Fiji software.

### Statistical Analysis

Graphpad Prism software 9.4.1was used for data organization, analysis, and visualization. Significance analyses between two groups were performed using Two‐tailed, nonparametric Mann Whitney t test. Multiple group testing was performed using one‐way ANOVA with Holm‐Šídák's (for normally distributed data) or nonparametric Kruskal‐Wallis with Dunn's multiple comparison post‐hoc testing. *p*‐values and samples size are indicated in the figures and figure legends, respectively.

## Conflict of Interest

The authors declare no conflict of interest.

## Author Contributions

A.A.H. and J.G.S. conceived the study and designed the experiments. A.A.H., B.N., M.B., and N.G. developed experimental protocols and conducted experiments. A.A.H. analyzed and interpreted the data and wrote the manuscript. J.G.S. acquired funding, supervised the project, interpreted the data, and revised the manuscript. All authors approved the manuscript. The funding sources had no influence on data collection, design, analysis, interpretation; or any aspect pertinent to the study.

## Supporting information

Supporting Information

## Data Availability

The data that support the findings of this study are available from the corresponding author upon reasonable request.
