# Supplementary material for: The Stiffness‐Sensitive Transcriptome of Human Tendon Stromal Cells
Source: Adv Healthc Mater. 2023 Jan 20;12(7):2101216. doi: 10.1002/adhm.202101216 (PMC11468939; doi:10.1002/adhm.202101216)
Supplement: Supplementary file 1 — Supporting Information [file ADHM-12-2101216-s001.pdf]

ADVANCED HEALTHCARE MATERIALS

Supporting Information

for *Adv. Healthcare Mater.*, DOI 10.1002/adhm.202101216

The Stiffness-Sensitive Transcriptome of Human Tendon Stromal Cells

*Amro A. Hussien, Barbara Niederoest, Maja Bollhalder, Nils Goedecke and Jess G. Snedeker**

Supplementary Information

The stiffness-sensitive transcriptome of human tendon stromal cells

*Amro A. Hussien, Barbara Niederoest, Maja Bollhalder, Nils Goedecke, Jess G. Snedeker**

Dr. A. A. Hussien, B. Niederoest, M. Bollhalder, Dr. N. Goedecke, Dr. J. G. Snedeker
Institute for Biomechanics, ETH Zurich, Zurich, 8092, Zurich, Switzerland.
Balgrist University Hospital, University of Zurich, Zurich, 8008, Zurich, Switzerland.
E-mail: snedeker@ethz.ch (Jess G. Snedeker)

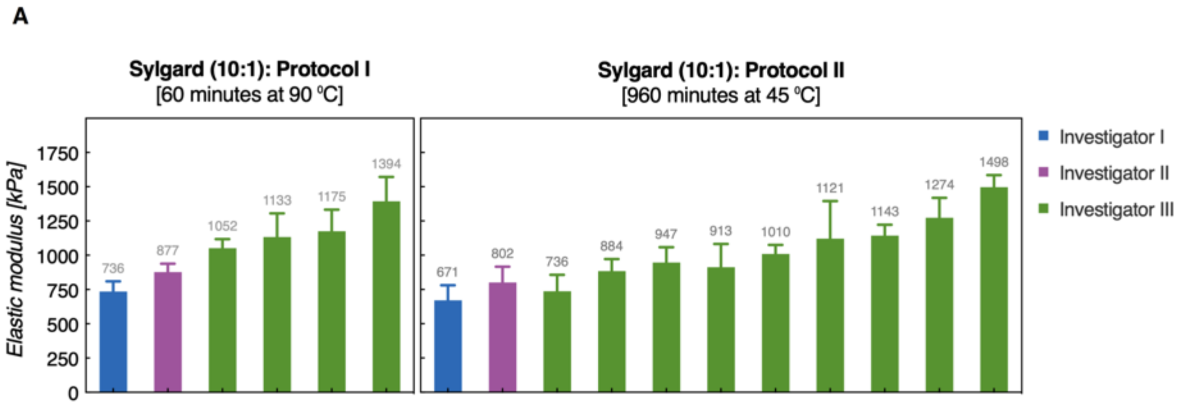

Supplementary figure S1 | Characterization of Sylgard 184® [10:1] mixing ratio.
(A) Material characterization of Sylgard 184® (10:1) mechanical properties (*E.* modulus) for the indicated heat curing protocols. Substrate properties were measured by micro-indentation using a calibrated piezoresistive Femto-Tools™ probe. Bars depict the mean ± SD. Plotted values indicate the mean.

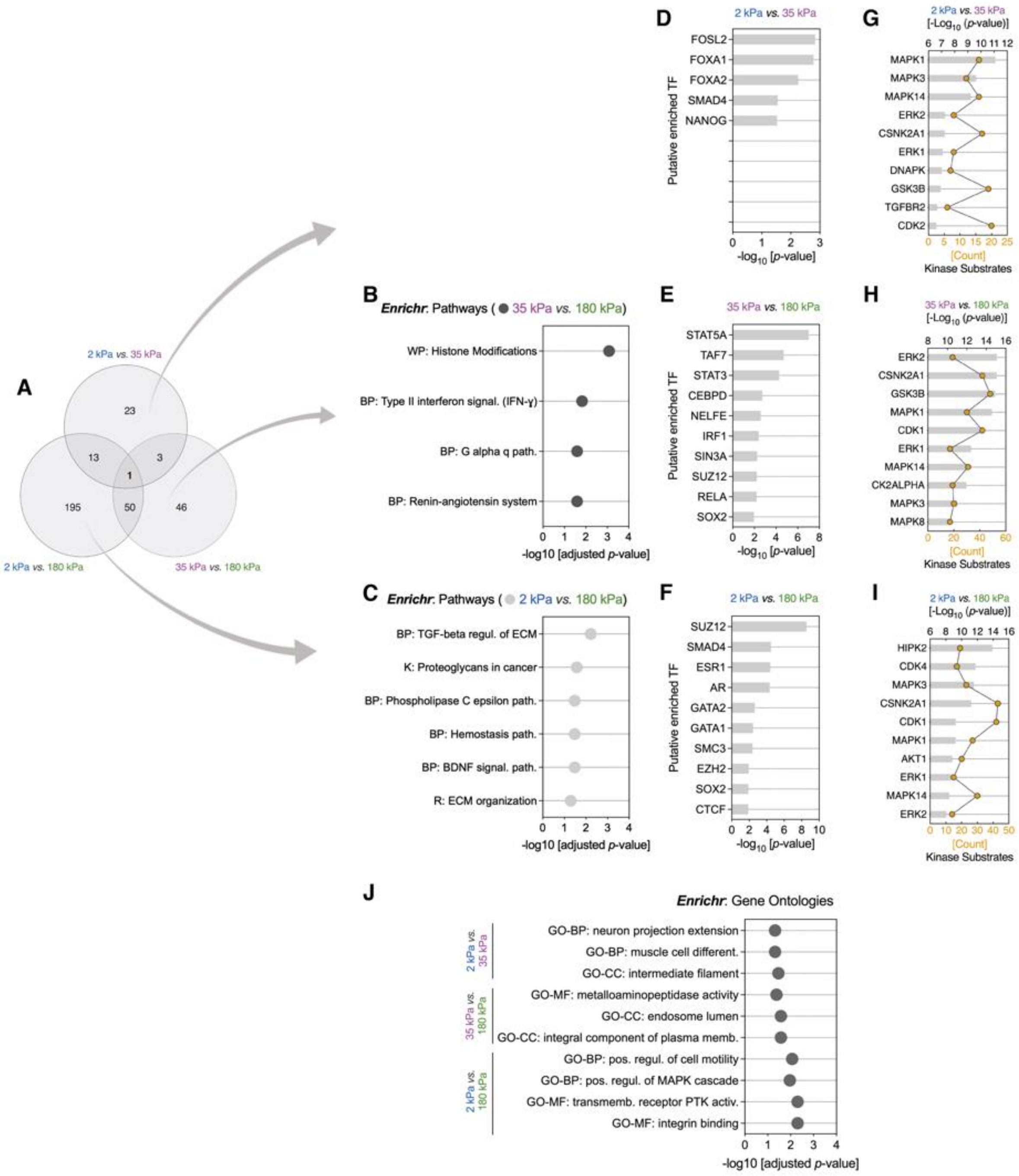

Supplementary figure S2 | Stiffness-specific enriched pathways and inferred upstream kinases and transcription factors.

(A) Venn diagram depicting the overlapped significantly expressed genes (DEGs) between different stiffness comparisons. Unique genes to each stiffness comparison (*i.e.* stiffness-specific genes) were used for downstream analysis. **(B-C)** Top enriched pathways for each stiffness-specific comparison; (H) 35kPa *vs.* 180kPa, (E) 2kPa *vs.* 180kPa. Enrichment analysis was performed in *Enrichr*. **(D-F)** Bar plots show the predicted top 5-10 most significantly enriched transcription factors upstream of the stiffness-specific DEGs. Predicted TFs are sorted by significance level (adjusted p-value < 0.05). **(G-I)** Bar plots indicate the predicted top 10 most significantly enriched kinases upstream of the stiffness-specific DEGs. Predicted kinases are ranked by significance level (adjusted p value < 0.05). TF and kinases were predicted using the TF and Kinase Enrichment Analysis modules of the *Expression2Kinase* tool. **(J)** Stiffness-specific top enriched GO terms. Enrichment analysis was performed in *Enrichr*. Significance achieved at q-value < 0.05.

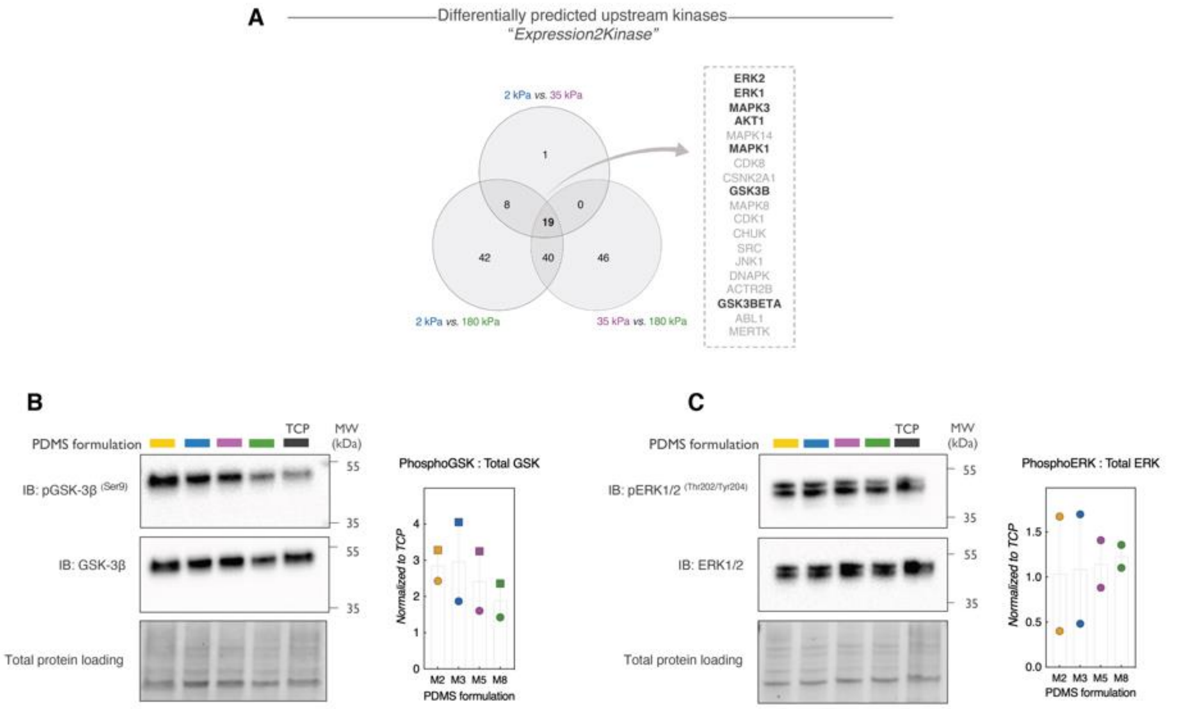

Supplementary figure S3 | Experimental validation of predicted stiffness-sensitive kinases.
(A) Venn diagram depicting the overlapped predicted upstream kinases between the different stiffness comparisons using Expression2Kinase tool. Experimentally validated hits are highlighted in bold. **(B - C)** Experimental immunoblot validation of (B) GSK-3β$^{(Ser9)}$ and (C) ERK1/2$^{(Thr202/Tyr204)}$ kinase phosphorylation in response to substrate stiffness. Data are from two independent biological donors. All data is shown as fold change to TCP.

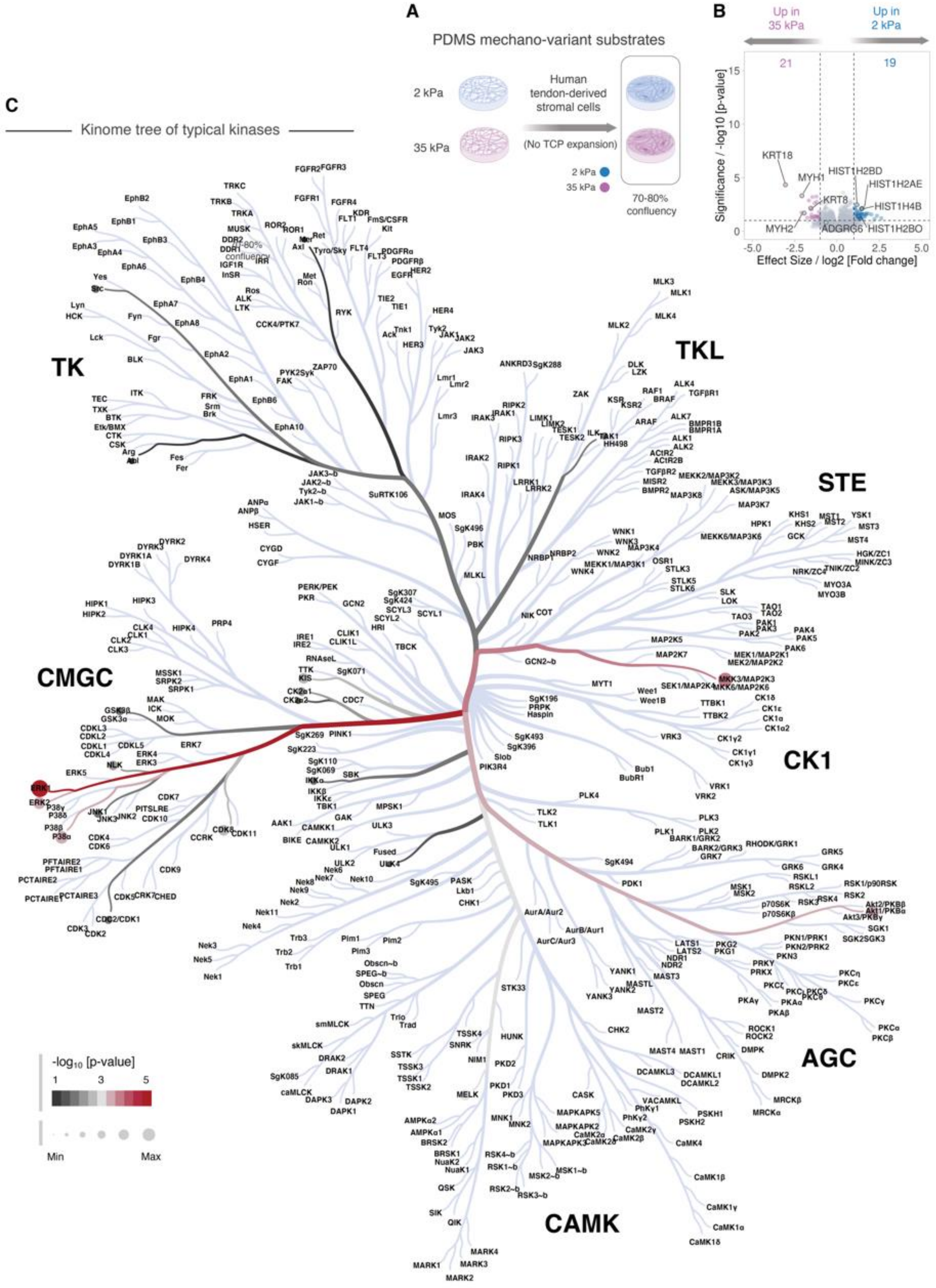
A
PDMS mechano-variant substrates
2 kPa
35 kPa
Human tendon-derived stromal cells
(No TCP expansion)
2 kPa
35 kPa
70-80% confluency
B
Up in 35 kPa
Up in 2 kPa
21
19
Significance / -log10 [p-value]
Effect Size / log2 [Fold change]
KRT18
MYH1
HIST1H2BD
HIST1H2AE
KRT8
HIST1H4B
MYH2
ADGRG6
HIST1H2BO
C
Kinome tree of typical kinases
TK
TKL
STE
CK1
AGC
CAMK
CMGC
-log10 [p-value]
1
3
5
Min
Max

Supplementary figure S4 | Predicted key signaling kinases upstream of the DEGs in 2 kPa *vs.* 35 kPa PDMS.

(A) Schematic of the experimental conditions. **(B)** RNA-seq volcano plot of DEGs. Horizontal line corresponding to *p* value ≤ 0.01 and vertical lines are at a cutoff of log2 [Fold change] ± 1. The same panel also appears in Figure 3 B. **(B)** Kinome tree dendrogram mapping of all the enriched upstream protein kinases in 2 kPa *vs.* 35 kPa PDMS. Circles' color and size encode the enrichment significance value, with red color reflecting higher significance. Blue branches depict "no value" background.

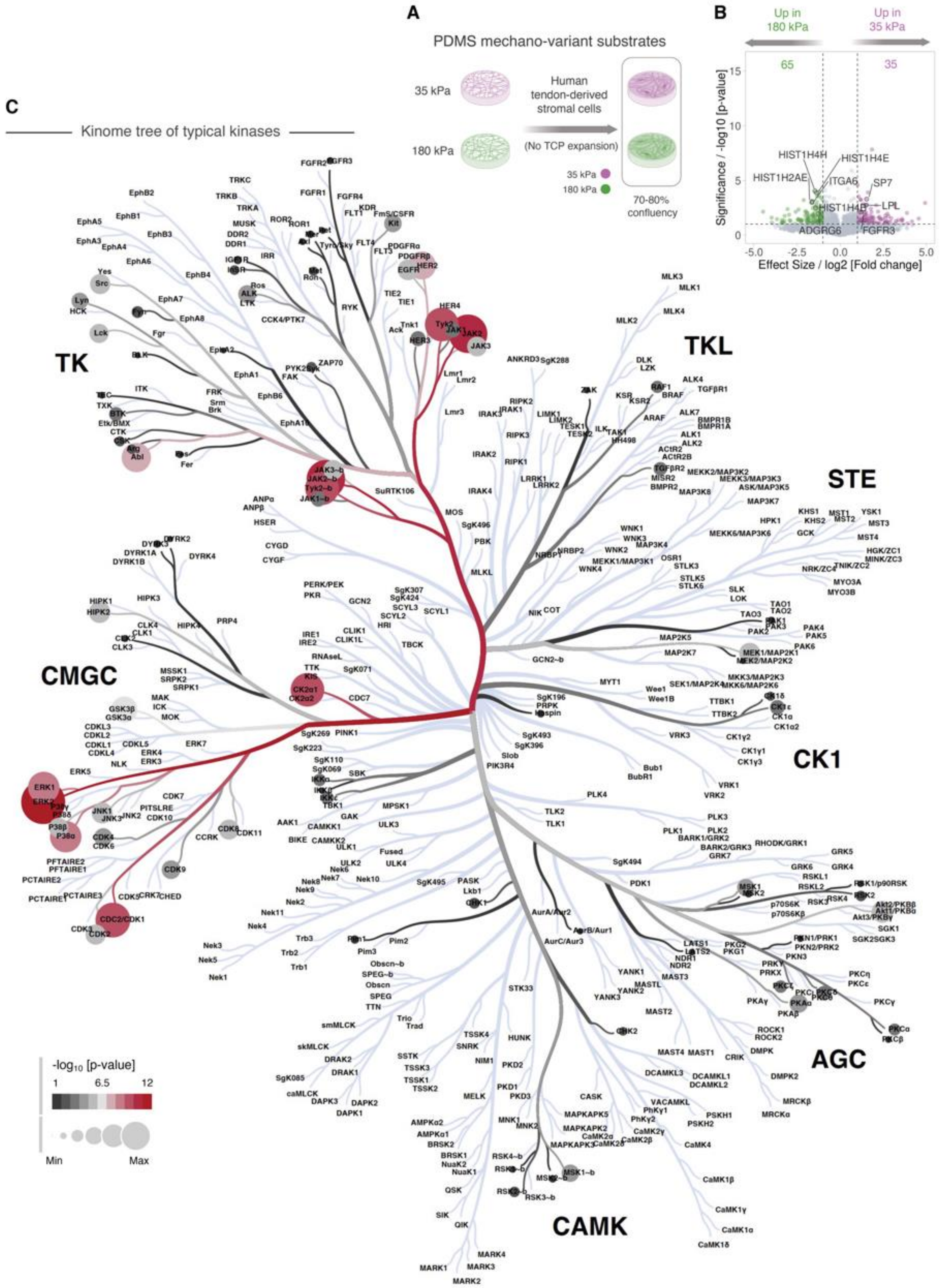

Supplementary figure S5 | Predicted key signaling kinases upstream of the DEGs in 35 kPa *vs.* 180 kPa PDMS.

(A) Schematic of the experimental conditions. **(B)** RNA-seq volcano plot of DEGs. Horizontal line corresponding to *p* value ≤ 0.01 and vertical lines are at a cutoff of log2 [Fold change] ± 1. The same panel also appears in Figure 3 B. **(B)** Kinome tree dendrogram mapping of all the enriched upstream protein kinases in 35 kPa *vs.* 180 kPa PDMS. Circles' color and size encode the enrichment significance value, with red color reflecting higher significance. Blue branches depict "no value" background.

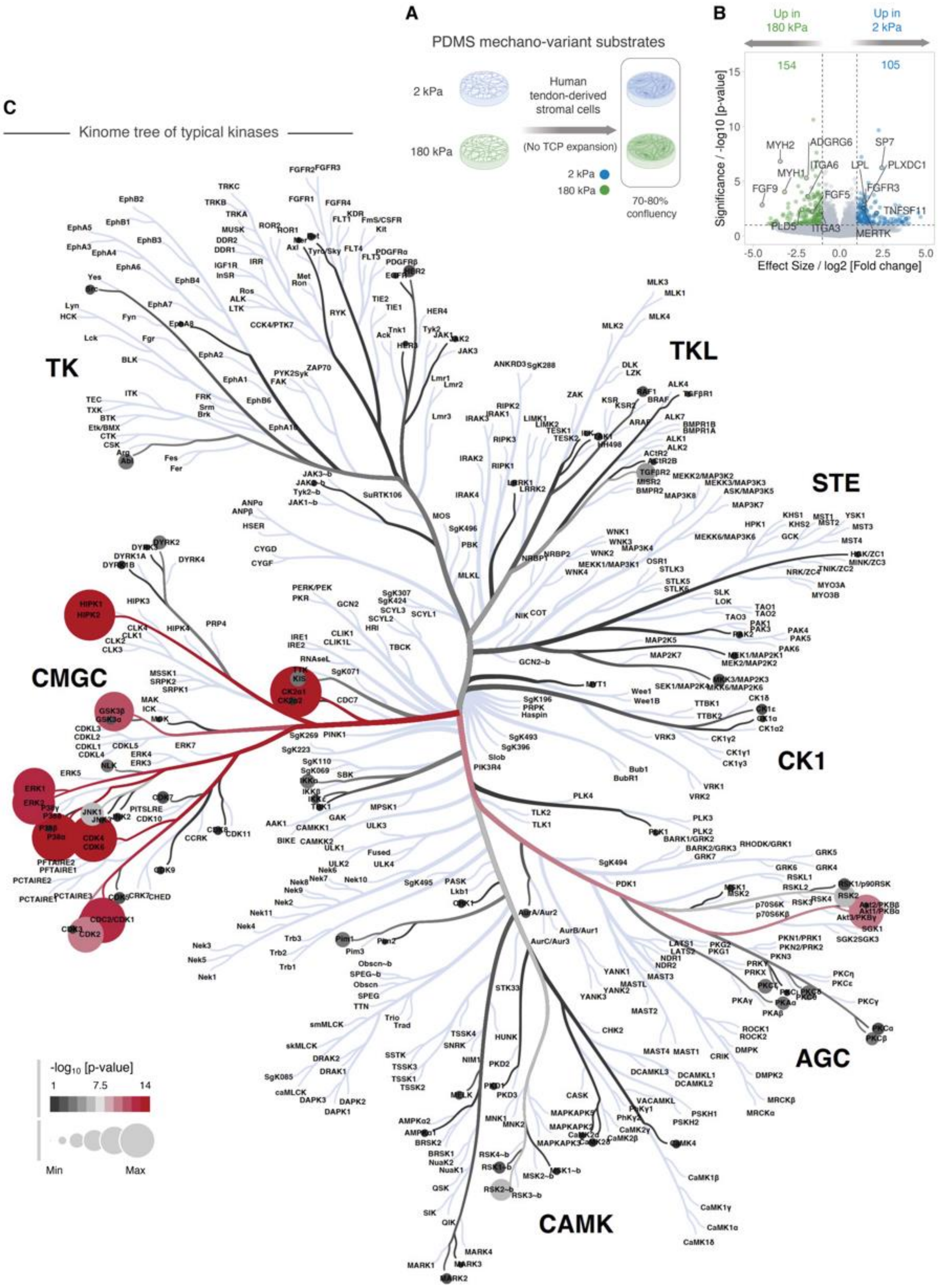

Supplementary figure S6 | Predicted key signaling kinases upstream of the DEGs in 2 kPa *vs.* 180 kPa PDMS.

(A) Schematic of the experimental conditions. **(B)** RNA-seq volcano plot of DEGs. Horizontal line corresponding to *p* value ≤0.01 and vertical lines are at a cutoff of log2 [Fold change] ± 1. The same panel also appears in Figure 3 B. **(B)** Kinome tree dendrogram mapping of all the enriched upstream protein kinases in 2 kPa *vs.* 180 kPa PDMS. Circles' color and size encode the enrichment significance value, with red color reflecting higher significance. Blue branches depict "no value" background.

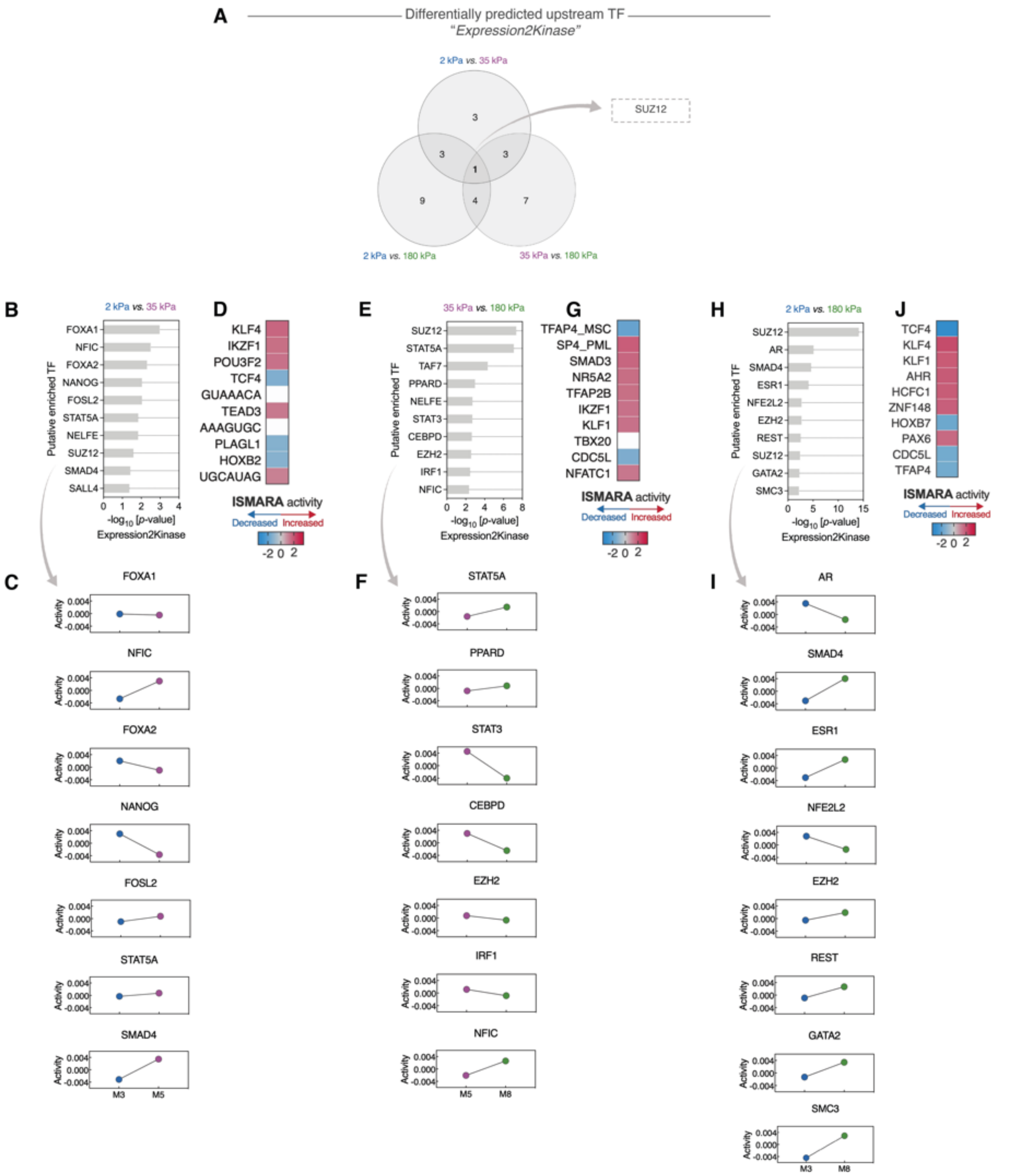
A
Differentially predicted upstream TF
"Expression2Kinase"
2 kPa vs. 35 kPa
SUZ12
2 kPa vs. 180 kPa
35 kPa vs. 180 kPa
B
D
E
G
H
J
C
F
I
Putative enriched TF
-log10 [p-value]
Expression2Kinase
ISMARA activity
Decreased
Increased
Activity
M3
M5
M8

Supplementary figure S7 | Predicted upstream transcription factors (TFs) using Expression2Kinase and ISMARA tools.

(A) Venn diagram depicting the overlapped predicted upstream TFs between the different stiffness comparisons using Expression2Kinase tool. **(B)** Bar plot shows the Expression2Kinase predicted top 10 most significantly enriched transcription factors upstream of the DEGs in (2 *vs.* 35 kPa) comparison. Predicted TFs are sorted by significance level (adjusted p-value < 0.05). **(C)** ISMARA TF activity of Expression2Kinase hits in (B). **(D)** ISMARA inferred regulatory motif analysis of predicted TFs activity at proximal promoter regions in the (2 *vs.* 35 kPa) comparison. **(E)** Bar plot depicts the Expression2Kinase predicted top 10 most significantly enriched transcription factors upstream of the DEGs in (35 *vs.* 180 kPa) comparison. Predicted TFs are sorted by significance level (adjusted p-value < 0.05). **(F)** ISMARA TF activity of Expression2Kinase hits in (E). **(G)** ISMARA inferred regulatory motif analysis of predicted TFs activity at proximal promoter regions in the (35 *vs.* 180 kPa) comparison. **(H)** Bar plot shows the Expression2Kinase predicted top 10 most significantly enriched transcription factors upstream of the DEGs in (2 *vs.* 180 kPa) comparison. Predicted TFs are sorted by significance level (adjusted p-value < 0.05). **(I)** ISMARA TF activity of Expression2Kinase hits in (H). **(J)** ISMARA inferred regulatory motif analysis of predicted TFs activity at proximal promoter regions in the (2 *vs.* 180 kPa) comparison. Panels (B, E and H) also appear in Figure 2, and are included here to facilitate readability of the graph.

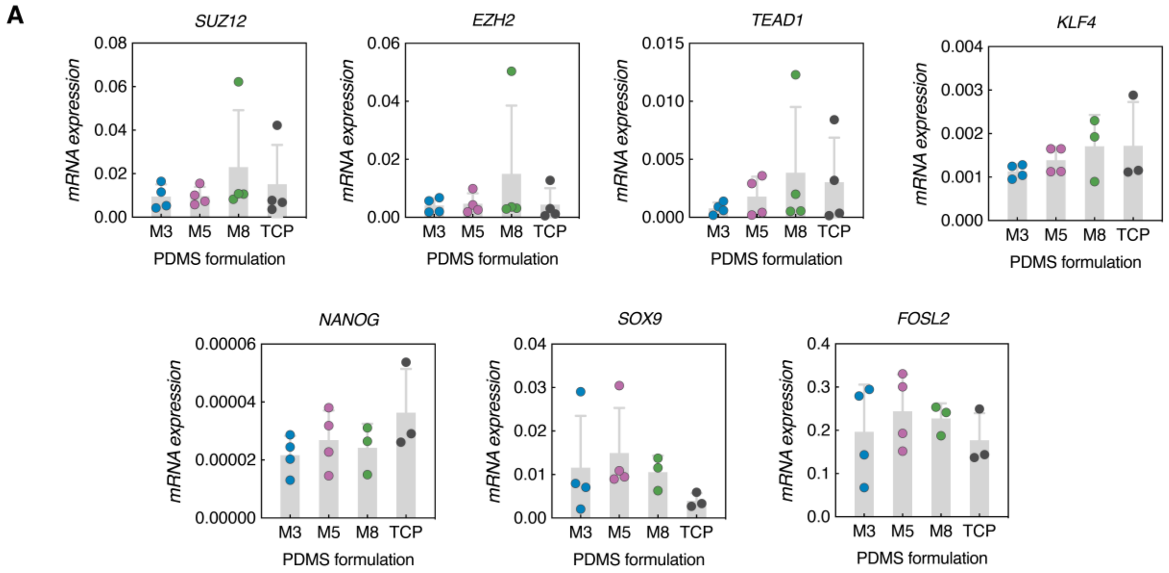

Supplementary figure S8 | Computationally predicted transcription factors (TFs) are expressed in tendon stromal cells.

(A) mRNA expression profiles of some of the computationally predicted transcription factors (TFs) in tendon stromal cells seeded on mechano-variant substrates. Gene expression is measured by RT-qPCR at 48 hours. (N = 4 independent biological donors). Data points represent ΔCt values. Expression of individual genes shown normalized to *RPL13A* and *GAPDH* reference genes. Bars depict the mean ± SD.

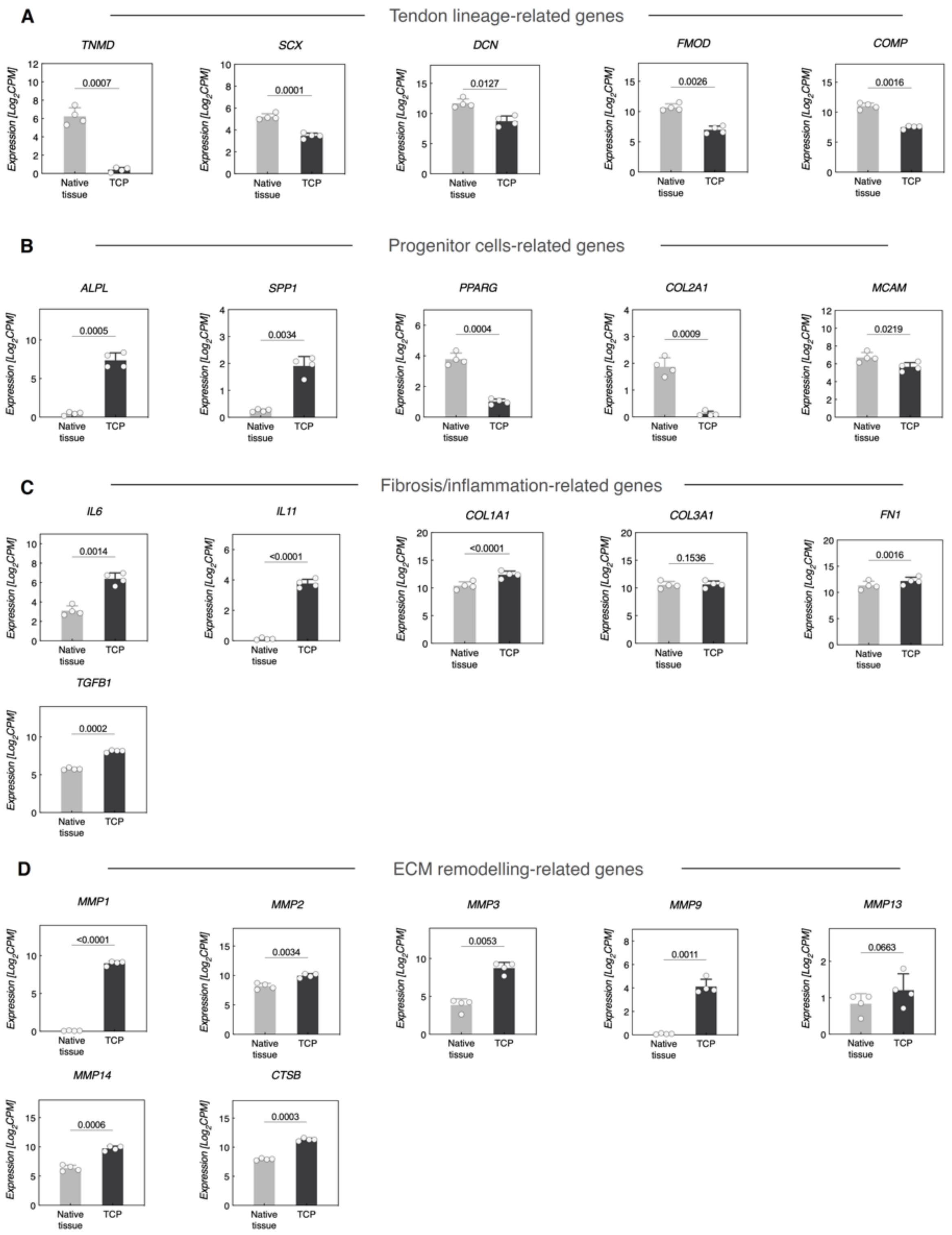

Supplementary figure S9 | Phenotypic drift is evident in TCP conditioned tendon fibroblasts.
RNA-Seq expression values of **(A)** Tendon lineage-related genes, **(B)** Progenitor cells differentiation markers, **(C)** Fibrosis/inflammation-related genes, and **(D)** ECM remodeling-related genes. Control represents native tendon tissue. (N = 4 independent biological donors).

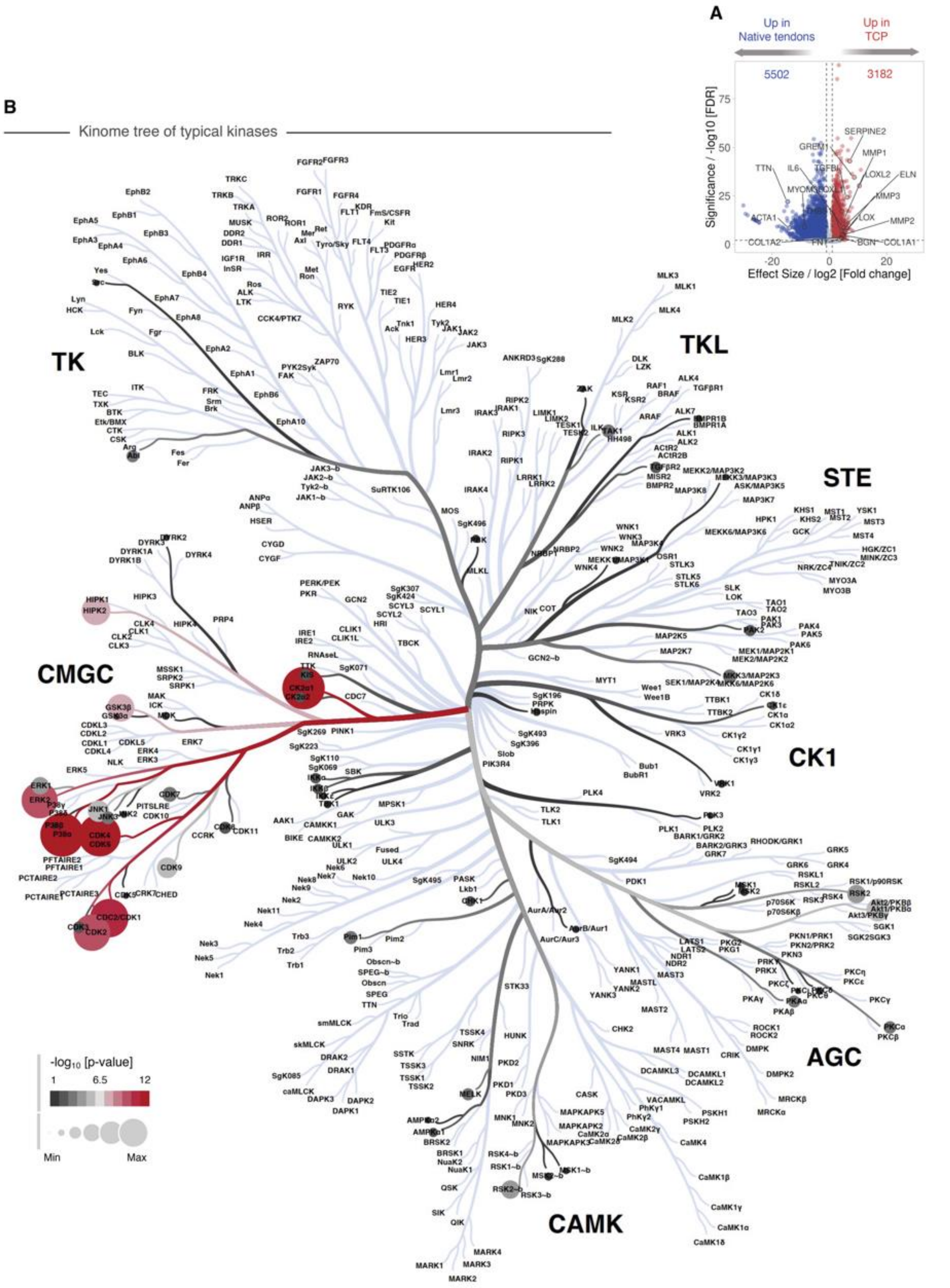

Supplementary figure S10 | Predicted key signaling kinases upstream of the DEGs in TCP *vs.* Native tendons.

(**A**) RNA-seq volcano plot of DEGs. Horizontal line corresponding to an FDR ≤0.01 and vertical lines are at a cutoff of log2[Fold change] ± 1. The same panel also appears in Figure 2C. **(B)** Kinome tree dendrogram mapping of all the enriched upstream protein kinases in TCP *vs.* Native tendons. Circles' color and size encode the enrichment significance value, with red color reflecting higher significance. Blue branches depict "no value" background.

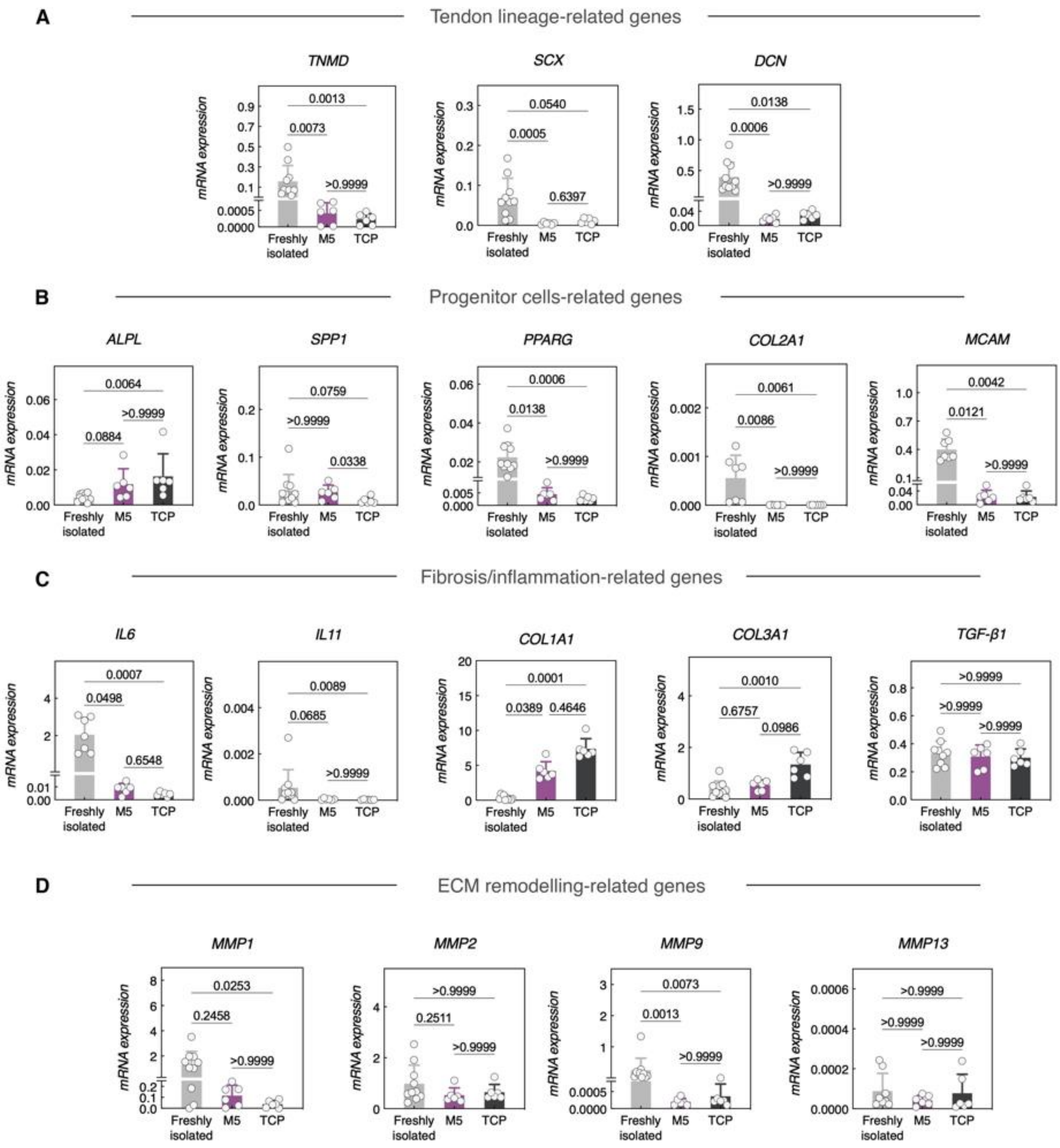

Supplementary figure S11 | Gene expression profiles of freshly isolated cells and cells conditioned to M5 (35 kPa) or TCP.

RT-qPCR values of **(A**) Tendon lineage-related genes, **(B)** Progenitor cells differentiation markers, **(C)** Fibrosis/inflammation-related genes, and **(D)** ECM remodeling-related genes. (N = 10 for freshly isolated cells, N = 6 for M5 (35 kPa) and TCP from independent biological donors)

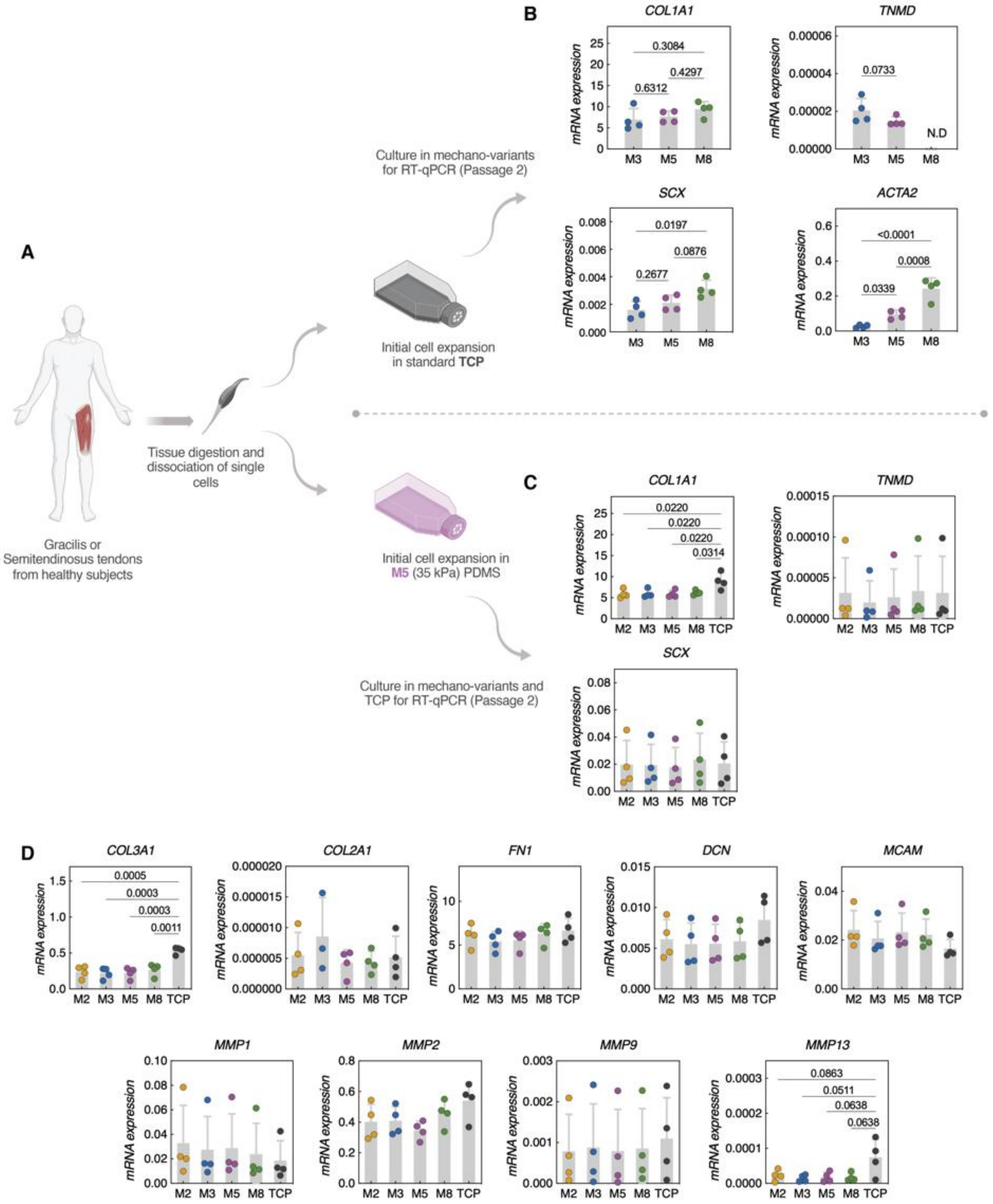

Supplementary figure S12 | The impact of initial substrate priming on mRNA expression of tendon stromal cells. (A) Freshly isolated tendon stromal cells were initially expanded on **(B)** TCP or **(C-D)** 35 kPa PDMS for one passage before transfer to mechano-variant substrates at the subsequent passage. (N = 4 independent biological donors).

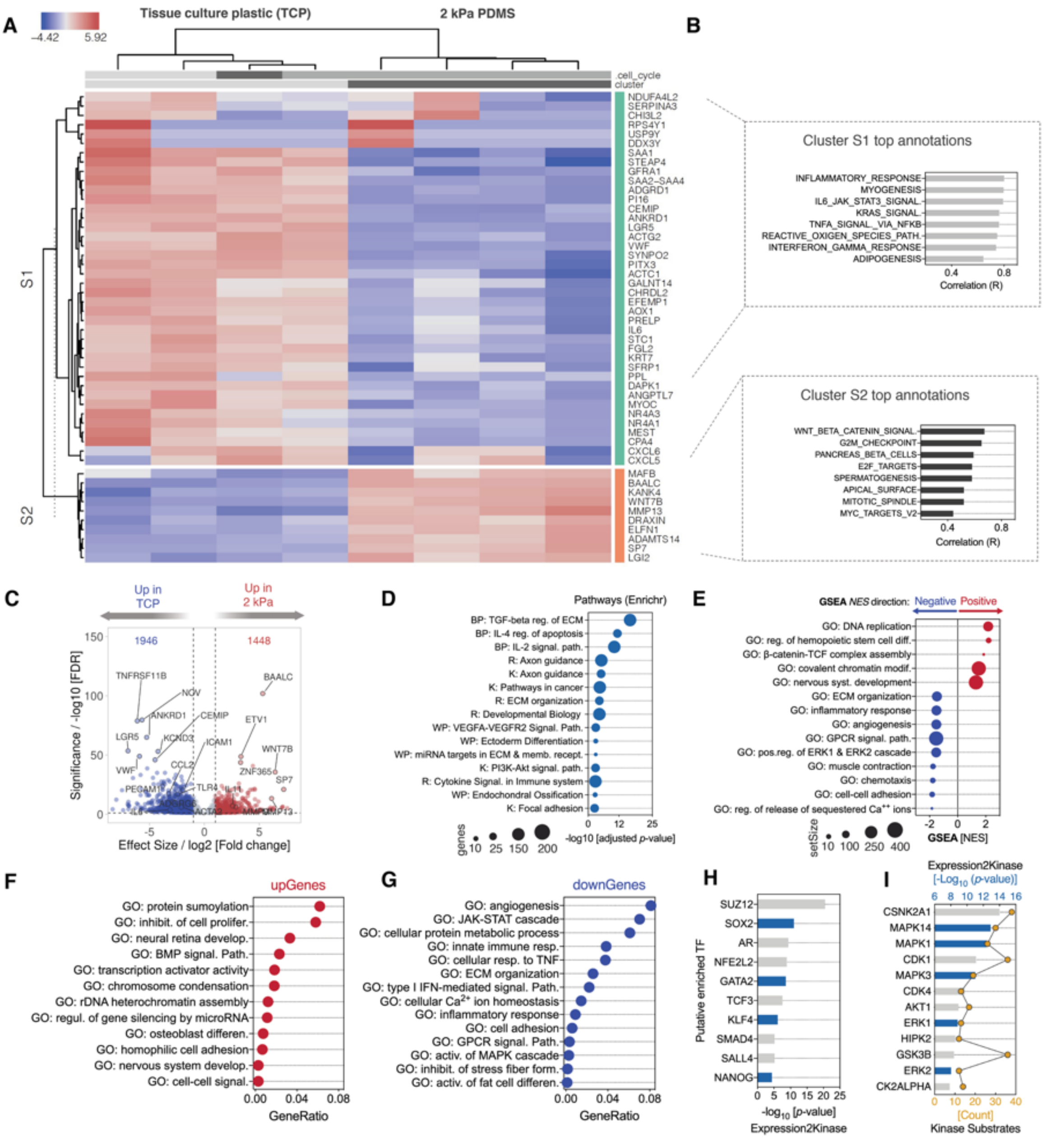

Supplementary figure S13 | Transcriptome analysis of tendon-derived stromal cells on 2 kPa soft substrates *vs.* TCP.

(A) Heatmap of gene-level hierarchical clustering of the top 50 differentially expressed genes (DEG) in tendon stromal fibroblasts cultured on soft PDMS substrates (*E.* 2 kPa) *vs.* tissue culture plastic (TCP). Columns represent individual samples (N= 4 biological replicates from different donors). Blue denotes downregulated genes; red denotes upregulated genes. **(B)** Functional annotation of S1 and S2 gene clusters in the heatmap. Bar plots depict the fisher-weighted, average correlations of the cluster with the annotation terms queried against MSigDB Hallmark database. **(C)** RNA-seq volcano plot of DEGs of 2 kPa conditioned human tenocytes relative to TCP control. Colored dots show the 3,394 significantly expressed genes, as determined by *DESeq2* methods, with the horizontal line corresponding to an FDR ≤0.05 and vertical lines are at a cutoff of log2[Fold change] ± 1. **(D)** Enriched pathways analysis of a subset of DEGs using *Enrichr* queried against BioPlanet 2019, Reactome and WikiPathways 2019 Human databases. All hits had an adjusted *p*-value < 0.05. **(E)** Pre-ranked Gene Set Enrichment Analysis (*GSEA*) of positively and negatively enriched biological processes by Normalized Enrichment Score (NES) in 2 kPa cultured cells (Adjusted *p*-value < 0.05). **(F-G)** Overrepresentation analysis (ORA) of Biological Processes GO terms of upregulated (F) and downregulated (G) DEGs. Analysis was performed using a hypergeometric over-representation test against the GO database,

with significance cutoff at *q*-value < 0.05. **(H)** Bar plot depicts the predicted top 10 most significantly enriched transcription factors upstream of the DEGs. Predicted TFs are sorted by significance level (adjusted *p*-value < 0.05). **(I)** Top 10 kinases upstream of predicted TFs in (F) were identified using the Kinase Enrichment Analysis module of the *Expression2Kinase* pipeline.

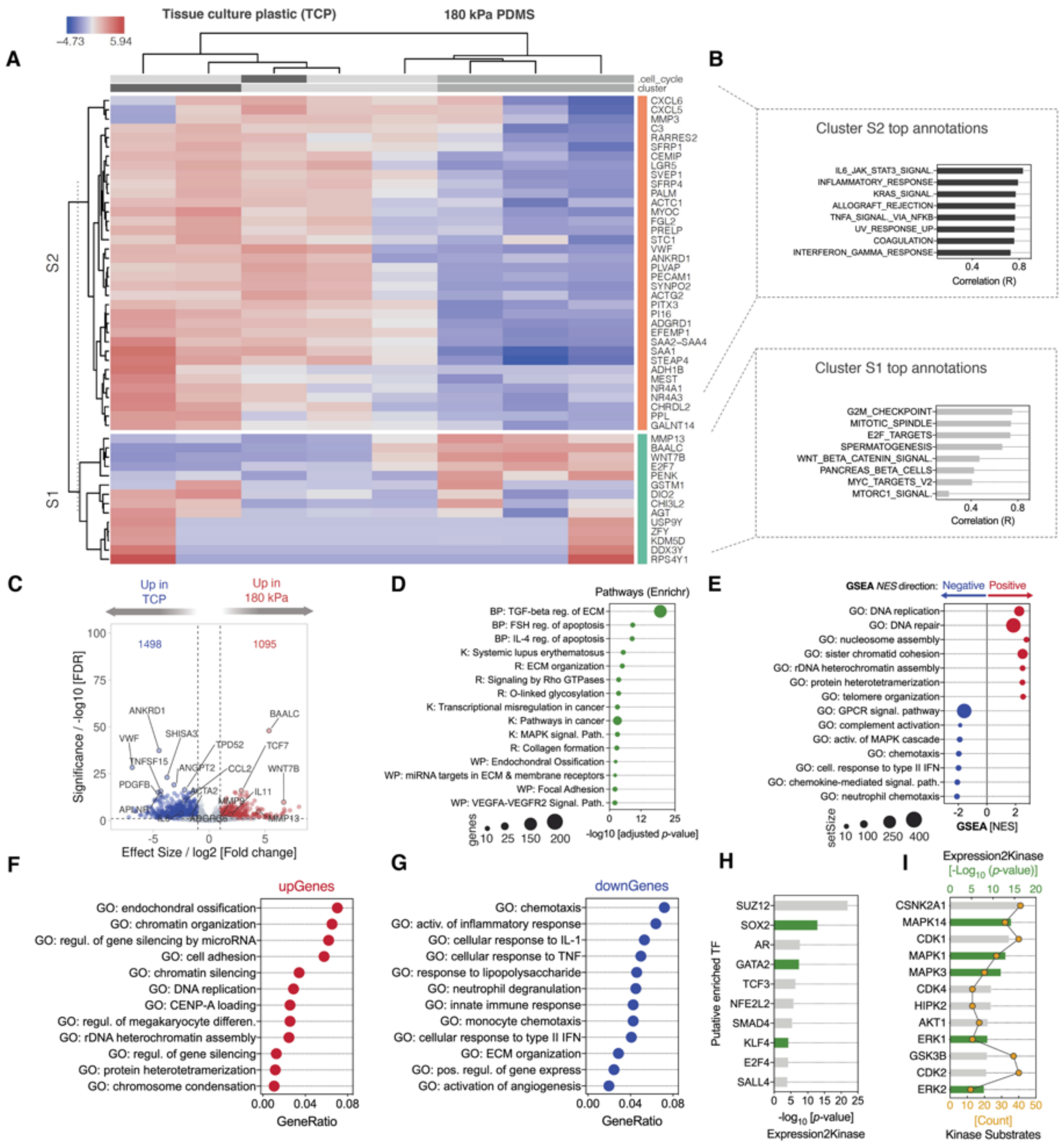

Supplementary figure S14 | Transcriptome analysis of tendon-derived stromal cells on 180 kPa stiff substrates *vs.* TCP.

(A) Heatmap of gene-level hierarchical clustering of the top 50 differentially expressed genes (DEG) in tendon stromal fibroblasts cultured on stiff PDMS substrates (*E.* 180 kPa) *vs.* tissue culture plastic (TCP). Columns represent individual samples (N= 4 biological replicates from different donors). Blue denotes downregulated genes; red denotes upregulated genes. **(B)** Functional annotation of S1 and S2 gene clusters in the heatmap. Bar plots depict the fisher-weighted, average correlations of the cluster with the annotation terms queried against MSigDB Hallmark database. **(C)** RNA-seq volcano plot of DEGs of 180 kPa conditioned human tenocytes relative to TCP control. Colored dots show the 2,593 significantly expressed genes, as determined by *DESeq2* methods, with the horizontal line corresponding to an FDR ≤0.05 and vertical lines are at a cutoff of log2[Fold change] ± 1. **(D)** Enriched pathways analysis of a subset of DEGs using *Enrichr* queried against BioPlanet 2019, Reactome and WikiPathways 2019 Human databases. All hits had an adjusted *p*-value < 0.05. **(E)** Pre-ranked Gene Set Enrichment Analysis (*GSEA*) of positively and negatively enriched biological processes by Normalized Enrichment Score (NES) in 180 kPa cultured cells (Adjusted *p*-value < 0.05). **(F-G)** Overrepresentation analysis (ORA) of Biological Processes GO terms of upregulated (F) and downregulated (G) DEGs. Analysis was performed using a hypergeometric over-representation test against the GO database, with significance cutoff at

q-value < 0.05. **(H)** Bar plot depicts the predicted top 10 most significantly enriched transcription factors upstream of the DEGs. Predicted TFs are sorted by significance level (adjusted *p*-value < 0.05). **(I)** Top 10 kinases upstream of predicted TFs in (F) were identified using the Kinase Enrichment Analysis module of the *Expression2Kinase* pipeline.

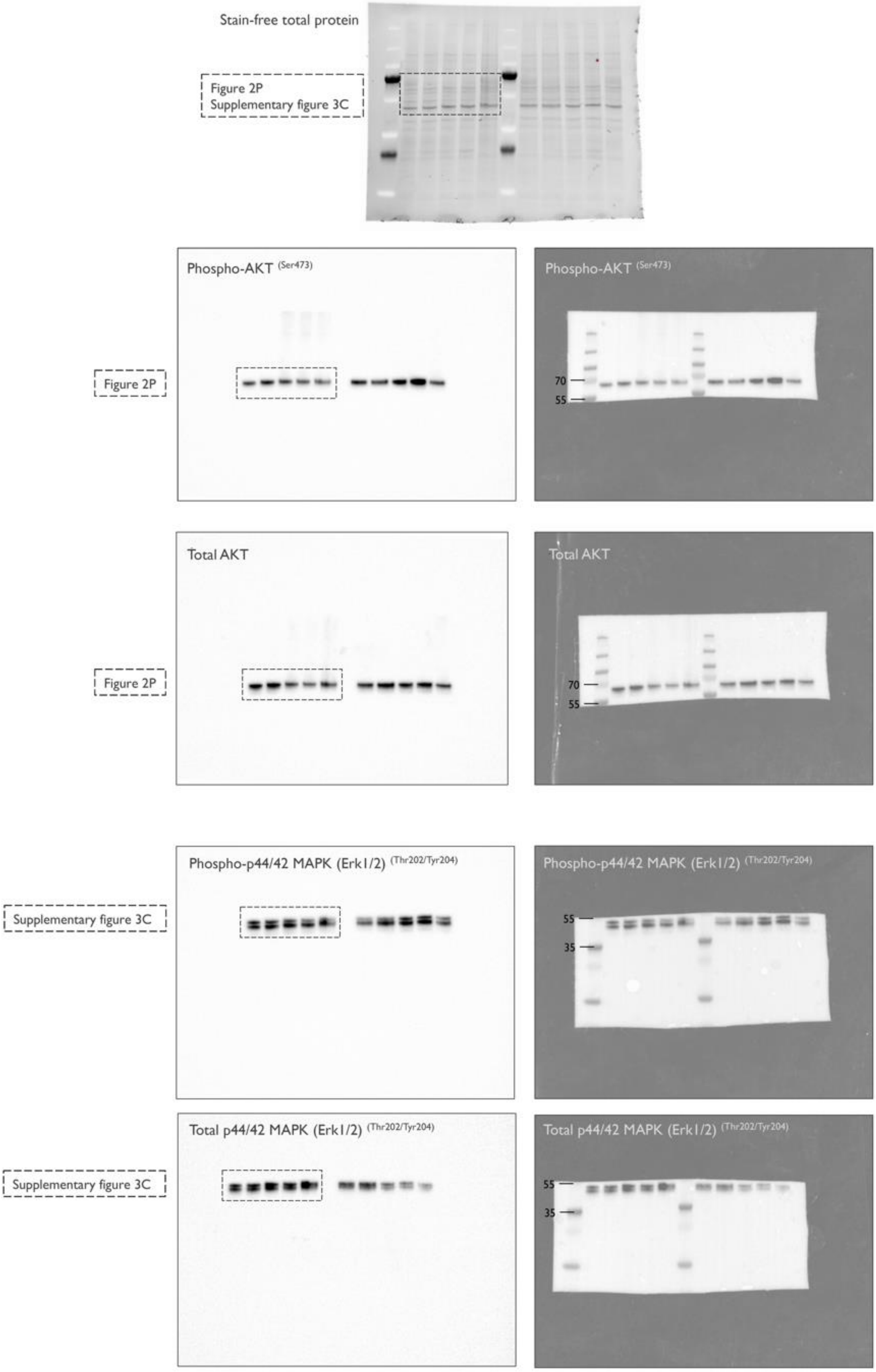
Stain-free total protein
Figure 2P
Supplementary figure 3C
Phospho-AKT (Ser473)
Figure 2P
Phospho-AKT (Ser473)
70
55
Total AKT
Figure 2P
Total AKT
70
55
Phospho-p44/42 MAPK (Erk1/2) (Thr202/Tyr204)
Supplementary figure 3C
Phospho-p44/42 MAPK (Erk1/2) (Thr202/Tyr204)
55
35
Total p44/42 MAPK (Erk1/2) (Thr202/Tyr204)
Supplementary figure 3C
Total p44/42 MAPK (Erk1/2) (Thr202/Tyr204)
55
35

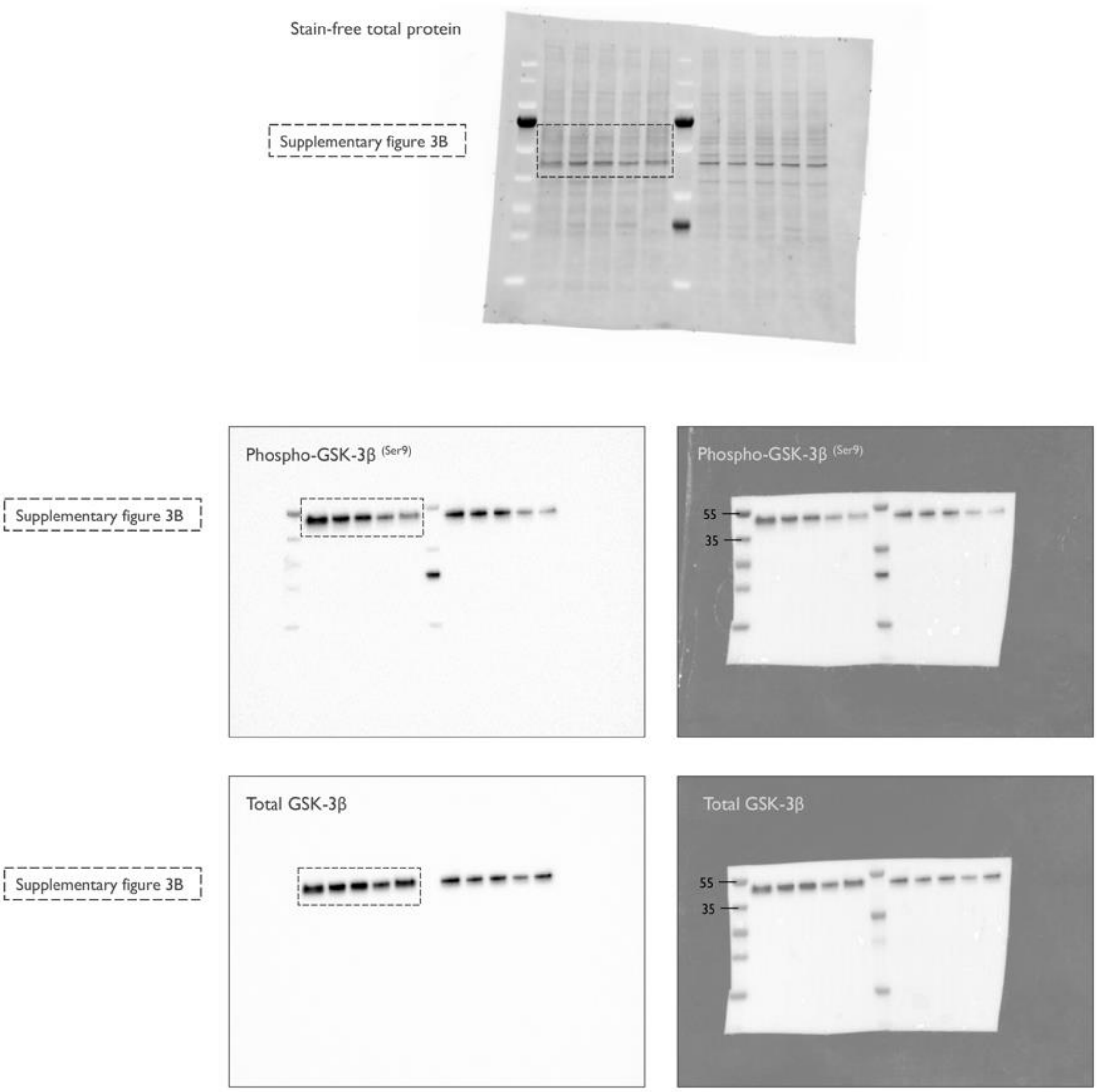

Supplementary figure S15 | Uncropped scans of western blot membranes.

Supplementary Table S1 | Patient donor details and demographics.

Healthy tissues were obtained from healthy donors undergoing autograft tendon transfer procedures for the surgical repair of anterior cruciate ligaments (ACL).

Tissue banking ID	Age	Sex	Tendon	Clinical info
326	27	F	Semitendinosus	ACL, Non-diabetic
936	27	M	Semitendinosus	ACL, Non-diabetic
964	27	M	Semitendinosus	ACL, Non-diabetic
1002	21	F	Semitendinosus	ACL, Non-diabetic
1010	30	M	Semitendinosus	ACL, Non-diabetic
1024	18	F	Semitendinosus	ACL, Non-diabetic
1320	23	M	Semitendinosus	ACL, Non-diabetic
1345	29	F	Gracilis	ACL, Non-diabetic
1369	25	M	Semitendinosus	ACL, Non-diabetic
1370	32	M	Gracilis	ACL, Non-diabetic
1347	39	F	Semitendinosus	ACL, Non-diabetic
1373	24	F	Gracilis	ACL, Non-diabetic
1313	37	M	Semitendinosus	ACL, Non-diabetic
1335	27	M	Semitendinosus	ACL, Non-diabetic
1342	31	F	Semitendinosus	ACL, Non-diabetic
1352	31	F	Semitendinosus	ACL, Non-diabetic

Supplementary Table S2 | List of primary and secondary antibodies

Antibody	Source	Dilution	Identifier
Phospho-Akt (Ser473) (D9E) XP® Rabbit mAb	Cell Signaling Technology	1:1000	Cat# 4060, RRID:AB_2315049
Akt Antibody, Rabbit pAb	Cell Signaling Technology	1:1000	Cat# 9272, RRID:AB_329827
Phospho-GSK-3β (Ser9) (D85E12) XP® Rabbit mAb	Cell Signaling Technology	1:1000	Cat# 5558, RRID:AB_10013750
GSK-3β (D5C5Z) XP® Rabbit mAb	Cell Signaling Technology	1:1000	Cat# 12456, RRID:AB_2636978
Phospho-p44/42 MAPK (Erk1/2) (Thr202/Tyr204) Rabbit mAb	Cell Signaling Technology	1:1000	Cat# 9101, RRID:AB_331646
p44/42 MAPK (Erk1/2) Antibody, Rabbit pAb	Cell Signaling Technology	1:1000	Cat# 9102, RRID:AB_330744
Anti-Mouse IgG (H+L), highly cross adsorbed-Peroxidase antibody produced in goat	Sigma-Aldrich	1:20000	Cat# SAB3701073, RRID:AB_2819088
Anti-Rabbit IgG (H+L), highly cross adsorbed-Peroxidase antibody produced in goat	Sigma-Aldrich	1:20000	Cat# SAB3700878

Supplementary Table S3 | RT-qPCR TaqMan primers

Gene	Gene Description	Species	Assay ID	Assay
COL1A1	collagen type I alpha 1 chain	Human	Hs00164004_m1	TaqMan®
COL3A1	collagen type III alpha 1 chain	Human	Hs00943809_m1	TaqMan®
FN1	fibronectin 1	Human	Hs01549976_m1	TaqMan®
ACTA2	actin, alpha 2, smooth muscle, aorta	Human	Hs00426835_g1	TaqMan®
SUZ12	SUZ12 polycomb repressive complex 2 subunit	Human	Hs.PT.58.26825863	Integrated DNA Technologies
EZH2	enhancer of zeste 2 polycomb repressive complex 2 subunit	Human	Hs.PT.58.1924301	Integrated DNA Technologies
TEAD1	TEA domain transcription factor 1	Human	Hs.PT.58.22785339	Integrated DNA Technologies
KLF4	KLF transcription factor 4	Human	Hs.PT.58.45542593	Integrated DNA Technologies
NANOG	Nanog Homeobox	Human	Hs.PT.58.21480849	Integrated DNA Technologies
SOX9	SRY-box transcription factor 9	Human	Hs.PT.58.38984663	Integrated DNA Technologies
FOSL2	FOS like 2, AP-1 transcription factor subunit	Human	Hs.PT.58.27116056	Integrated DNA Technologies
TNMD	tenomodulin	Human	Hs00223332_m1	TaqMan®
SCX	scleraxis bHLH transcription factor	Human	Hs03054634_g1	TaqMan®
DCN	decorin	Human	Hs00754870_s1	TaqMan®
ALPL	alkaline phosphatase	Human	Hs.PT.56a.40555206	Integrated DNA Technologies
SPP1	secreted phosphoprotein 1	Human	Hs.PT.58.19252426	Integrated DNA Technologies
PPARG	peroxisome proliferator activated receptor gamma	Human	Hs.PT.58.25464465	Integrated DNA Technologies

Supplementary Table S3 | RT-qPCR TaqMan primers

Gene	Gene Description	Species	Assay ID	Assay
COL2A1	collagen type II alpha 1 chain	Human	Hs.PT.58.4107778	Integrated DNA Technologies
MCAM	melanoma cell adhesion molecule	Human	Hs.PT.58.40453650	Integrated DNA Technologies
IL6	interleukin 6	Human	Hs.PT.58.40226675	Integrated DNA Technologies
IL11	interleukin 11	Human	Hs.PT.58.50431822	Integrated DNA Technologies
TGFB1	transforming growth factor beta 1	Human	Hs00998133_m1	TaqMan®
MMP1	matrix metallopeptidase 1	Human	Hs00899658_m1	TaqMan®
MMP2	matrix metallopeptidase 2	Human	Hs01548727_m1	TaqMan®
MMP9	matrix metallopeptidase 9	Human	Hs00957562_m1	TaqMan®
MMP13	matrix metallopeptidase 13	Human	Hs00233992_m1	TaqMan®
